# Vehicle-Conditional Split-Conformal Calibration for Risk-Budgeted Sub-Second Proxy-Triggered Vehicle Instability Warnings from Past-Only Sensor Slices

**DOI:** 10.3390/s26082302

**Published:** 2026-04-08

**Authors:** Jinzhe Yang, Jianzheng Liu, Kai Tian, Yier Lin, Junxia Zhang

**Affiliations:** 1 College of Mechanical Engineering, Tianjin University of Science & Technology, Tianjin 300222, China; 2 Tianjin Customs District Industrial Products Safety and Technical Center, Tianjin 300041, China

**Keywords:** proxy-triggered warning, onboard sensor time series, vehicle instability warning, split-conformal calibration, Mondrian conformal prediction, runtime safety monitoring, false-alarm control

## Abstract

Emergency maneuvers can drive vehicles into severe instability regimes within sub-second time scales, motivating last-moment warning interfaces with auditable false-alarm budgets. We study a proxy-triggered imminent-recognition setting: given a 0.1 s past-only slice of onboard signals, decide whether a conservative physics-defined instability proxy will trigger within the next τ=0.2 s. The contribution is, therefore, a calibrated warning for a safety-relevant surrogate event, not a claim of predicting crashes or true instability outcomes directly. Because the corpus is terminal-phase aligned, the default causal monitor (w=d=0.1 s, k=2) is warnable on only 18.3% of event runs; we, therefore, report run-level effectiveness both overall and conditional on warnability. We learn a lightweight hazard scorer and convert its scores into an operator-facing alarm rule via split-conformal calibration on held-out negative slices, exposing a slice-level false-alarm budget α with finite-sample, one-sided control of the marginal slice-level false positive rate (FPR) on exchangeable negatives. To address fleet heterogeneity, we additionally calibrate vehicle-conditioned (Mondrian) thresholds, enabling per-vehicle risk budgeting without retraining separate models. On the held-out test split at τ=0.2 s, the scorer achieves AUPRC ≈0.251 against a base rate of 0.638%, AUROC ≈0.986, and ECE ≈0.034. After calibration at α=5%, realized slice-level FPR concentrates near the prescribed budget while slice-level TPR on imminent positives remains high (≈0.982). We explicitly separate this slice-level guarantee from empirical run-level metrics such as FAR_run_, EWR on warnable runs, and lead time, and we report dependence and shift diagnostics to delineate where the guarantee may degrade. The reported μ-sensitivity analyses concern run-level descriptor perturbation and omission rather than validation of a within-run friction estimator with temporal lag. The result is a transparent, risk-budgeted monitoring primitive for last-moment vehicle-stability warning under clearly stated exchangeability assumptions.

## 1. Introduction

Rare yet safety-critical failures—the “long tail” of driving—remain a central obstacle to dependable automated vehicles. When an emergency maneuver pushes a vehicle close to its handling limits, stability margins can collapse within sub-second time scales due to tire saturation, yaw divergence, and rapid load transfer. In such regimes, a practical warning monitor must satisfy two competing requirements at once: (i) raise an alarm early enough to be actionable by downstream control or fallback logic, and (ii) keep false alarms under explicit control so that the interface remains usable in practice.

**Figure 1:**
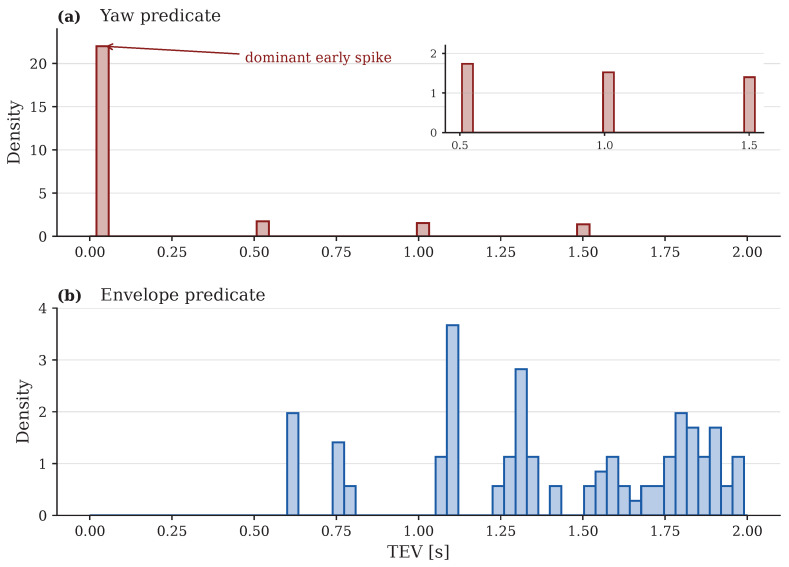
Terminal-phase alignment diagnostic. Histogram of proxy trigger times $t_{\mathrm{ev}}$ measured from episode start. A large fraction of events occur very early, limiting the number of causal pre-event slices available for warning.

Scope: proxy-triggered last-moment warning. This paper studies a last-moment pre-warning setting at the primary horizon τ=0.2 s: given only a 0.1 s past-only slice of onboard signals (10 Hz decision updates), decide whether a conservative physics-defined instability proxy will trigger within the next τ seconds. Our corpus is curated as short terminal-phase snippets around the onset of critical maneuvers, which means the available time-to-event support is inherently sub-second (Figure 1). Under the default causal monitor (w,d,k)=(0.1s,0.1s,2), only 18.3% of event runs are warnable, so run-level effectiveness is reported both overall and conditional on warnability. We therefore position the task as proxy-triggered imminent recognition, not as multi-second forecasting from benign driving and not as direct prediction of crash or rollover outcomes.

Why a proxy trigger is still safety-relevant.True instability outcomes are too sparse and too dependent on scenario-specific interventions to support the present study directly. Instead, we define a conservative surrogate event from three interpretable stability-margin predicates: friction-envelope exceedance, yaw-consistency breakdown, and elevated load-transfer ratio. Each predicate captures entry into a severe loss-of-margin regime that a downstream safety monitor would care about. Accordingly, the central claim of the paper is proxy-triggered warning calibration: we calibrate when to raise an alarm for this safety-relevant surrogate, and we interpret all reported metrics under that proxy semantics.

From scores to an auditable alarm rule.We learn a lightweight hazard scorer $f_{\tau }\left(x\right)\in \left(0,1\right)$ that maps each slice feature vector *x* to an imminent-instability score. A key objective is to convert scores into an operator-facing alarm rule with a slice-level risk knob α: the prescribed false-alarm budget on negative slices. To avoid ad hoc threshold selection, we adopt split-conformal calibration on held-out negative slices to select thresholds with finite-sample, one-sided control of the marginal slice-level false positive rate (FPR) on exchangeable negative slices [1,2]. Concretely, the calibrated alarm accepts a slice if $f_{\tau }\left(x\right)\leq t_{\alpha }^{\left(\tau \right)}$ and alarms if $f_{\tau }\left(x\right)>t_{\alpha }^{\left(\tau \right)}$, where $t_{\alpha }^{\left(\tau \right)}$ is an upper empirical quantile on calibration negatives.

Fleet heterogeneity via vehicle-conditioned calibration. A single global threshold can lead to uneven false-alarm behavior across heterogeneous platforms (e.g., differences in wheelbase, track width, center of gravity, or sensing characteristics). Rather than retraining separate models, we calibrate per-vehicle (Mondrian) thresholds that implement the same risk knob within each vehicle. This yields a simple fleet interface: one shared scorer and vehicle-conditional risk budgets.

Metric hierarchy for deployment.Rare-event warning papers often mix together several conceptually different metrics. We separate them explicitly. Slice-level FPR@α on negative slices is the sole formal guarantee. AUPRC, AUROC, ECE, and reliability curves diagnose slice-level model quality. FAR_run_, EWR on warnable runs, and lead time provide an empirical deployment view. Finally, blocked-bootstrap and subdomain-mismatch stress tests audit the exchangeability assumptions needed for conformal validity. Making this hierarchy explicit is important because each metric answers a different deployment question.

Empirical snapshot at τ=0.2 s.On the held-out test split, the hazard scorer achieves AUPRC 0.251 against a base rate of 0.638%, AUROC 0.986, and ECE 0.034 (Table 5). After global split-conformal calibration at α=5%, realized test FPR concentrates near the prescribed budget (FPR≈0.052) while preserving high slice-level TPR on imminent positives (TPR≈0.982). Vehicle-conditioned Mondrian calibration yields essentially the same aggregate operating point at τ=0.2 s (FPR≈0.053, TPR≈0.980) while providing a per-vehicle budgeting interface. To make the guarantee boundary explicit, we also report two diagnostics: a run-blocked bootstrap that keeps test FPR in the range 0.040–0.049 at nominal α=5%, and an intentional subdomain-mismatch stress test in which realized FPR can drift as high as 0.263 under severe speed-domain mismatch.

Contributions.

- Proxy-triggered imminent warning from short past-only slices. We formulate a τ=0.2 s proxy-triggered imminent warning task using only 0.1 s past-only slices, aligned with a 10 Hz operator interface and a clear last-moment interpretation under terminal-phase episode alignment.
- Risk-budgeted split-conformal calibration, globally and per vehicle. We turn learned hazard scores into an alarm rule with a slice-level false-alarm budget α, using split-conformal calibration on negative slices and vehicle-conditioned Mondrian thresholds to support fleet heterogeneity without per-vehicle retraining.
- A deployment-oriented evaluation hierarchy with explicit assumption diagnostics. We reorganize evaluation around a clear metric hierarchy, report warnability-aware run-level interpretation, and include dependence/shift diagnostics in the core evaluation so the validity boundary of the conformal interface is auditable rather than implicit.

Secondary audit artifacts—including the physics reference monitor, group-granularity ablations, and the optional path-margin diagnostic—are retained in the Supplementary Information.

## 2. Related Work

### 2.1. Vehicle Stability, Limit Handling, and Proxy-Based Warning Signals

Vehicle handling limits are governed by tire–road friction, load transfer, and coupled yaw–roll dynamics; standard references summarize friction-limited envelopes, yaw stability, and rollover-relevant geometry and indices [3,4,5]. Standardized aggressive maneuvers (e.g., severe lane-change obstacle avoidance) are frequently used to probe transient limit handling and benchmark near-limit response [6]. In autonomy and driver assistance, envelope notions are often used to constrain feasible trajectories, and safe-envelope formulations have been integrated with planning and tracking to keep the vehicle within model-based constraints [7].

Rollover and severe loss-of-control monitoring also has a long history of using load-transfer-based indices (LTR) and predictive variants. Predictive LTR formulations incorporate steering input and ESC-available signals to anticipate rollover risk before wheel lift-off [8,9], and robust controller designs explicitly enforce LTR bounds under uncertainty [10]. Recent controller-centric work continues this line with observer-based collision-free control under non-Gaussian noises and cloud-based collision-avoidance ACC under communication constraints [11,12]. Cooperative control between traffic signals and connected automated vehicles has likewise been explored using deep reinforcement learning at intersections [13]. Our paper is adjacent to these strands but not a substitute for them: rather than proposing a new controller, we build a lightweight monitoring interface that consumes short past-only slices and outputs a calibrated alarm with an explicit false-alarm budget.

### 2.2. Distribution-Free Calibration and Conformal Risk Control

Conformal prediction provides finite-sample, distribution-free guarantees by calibrating nonconformity scores using held-out data [1,2,14]. Split (inductive) conformal forms the practical backbone for scalable uncertainty quantification and predictive sets [15,16,17]. Beyond coverage, conformal methods have been extended to control monotone losses (risk control) [18] and to explicitly limit false positives under a user-defined tolerance in set-valued prediction [19]. We use this toolbox in a pragmatic alarm-calibration role: an arbitrary hazard score is converted into an alarm rule with a single operator-facing risk knob α that budgets false alarms on negative slices. This differs from selecting a threshold through ROC analysis or $F_{1}$ maximization, which does not itself implement an auditable risk budget.

### 2.3. Conditional (Mondrian) Conformal and Heterogeneity

A persistent deployment concern is that marginal guarantees can hide subpopulation failures. Work on conditional validity clarifies what is feasible when conditioning on side information and motivates practical conditional/Mondrian variants [20]. For classification with many labels and sparse per-label support, clustered class-conditional conformal methods aim to improve conditional behavior while retaining finite-sample guarantees [21]. In applied settings, Mondrian cross-conformal prediction has been used to handle imbalanced groups [22], while Mondrian-style conformal regressors and combinations of Mondrian inductive conformal predictors illustrate the classical granularity–power trade-off [23,24]. Recent analyses also study how far split conformal remains reliable under non-exchangeability, which is directly relevant whenever calibration and deployment streams exhibit temporal dependence or domain drift [25]. Our Scheme B uses vehicle-conditioned calibration to support per-vehicle auditing and budgeting under a shared scorer.

### 2.4. Runtime Monitoring, Safety Assurance, and Rare-Event Evaluation

Safety assurance in robotics and autonomous driving increasingly combines controller-side certificates with runtime monitoring and safety-case evidence [26,27]. Conformal methods have recently been used to tune warning systems with statistical guarantees [28] and to calibrate runtime monitors such as OOD detectors with controlled false positives [29]. On the autonomous-driving side, silent testing and SOTIF-oriented runtime identification of unknown unsafe scenarios highlight the value of explicit online validation interfaces [30], while recent reviews emphasize the need to organize SOTIF evidence and runtime safety arguments more systematically [31]. Finally, evaluating rare safety-critical failures is fundamentally sample-limited; the literature emphasizes the need for stress testing, accelerated evaluation, and honest rare-event metrics [32,33,34,35]. Our contribution fits this ecosystem as a compact, calibrated monitoring primitive: it does not replace controller synthesis or system-level assurance, but it exposes an auditable false-alarm interface and makes the validity boundary of that interface explicit.

## 3. Problem Setup and Data

### 3.1. Data Provenance and Episode Curation

Supplementary Table S1 summarizes dataset provenance and episode curation. The corpus is an anonymized controlled vehicle-dynamics benchmark of short single-maneuver episodes: each run is one 100 Hz terminal-phase snippet from a critical handling maneuver rather than a long naturalistic driving segment. The time-varying channels are onboard kinematic measurements, whereas vehicle signatures and scenario descriptors are attached as run-level metadata. Train/Val/Cal/Test partitioning is performed only after episode curation so that all slices from a run remain in a single split. The benchmark provider/program name is anonymized in the manuscript; the curation protocol relevant to statistical validity is specified here and in Supplementary Table S1, and the raw runs are available on request from the corresponding author. The resulting benchmark supports proxy-triggered terminal-phase warning evaluation; it should not be read as direct validation on naturalistic crash or rollover outcomes.

### 3.2. Terminal-Phase Episodes and Time Origin

Our corpus is curated for terminal-phase monitoring: each run is a short snippet extracted around the onset of a critical maneuver, so stability margins are already collapsing when the episode begins. We define the episode time origin t=0 at the start of each run and define event time $t_{\mathrm{ev}}$ as the first trigger of a conservative, physics-defined instability proxy (described below). As a diagnostic, Figure 1 shows that $t_{\mathrm{ev}}$ is heavily concentrated near the beginning of each run, implying that the available time-to-event support is inherently sub-second. In our data, the median event time is 0.02 s, and the mean is 0.20 s (Supplementary Table S2), which motivates focusing on the primary horizon τ=0.2 s as a last-moment warning setting rather than a multi-second forecast.

### 3.3. Slice-Based Proxy-Triggered Imminent Recognition Task

Vehicle logs are sampled at 100 Hz and segmented into past-only slices of duration 0.1 s with a stride of 0.1 s. For a slice ending at time *t*, we compute a feature vector x(t) from the measurements over (t−0.1s,t] and assign horizon-specific labels for alarm windows τ∈{0.2,0.4,0.8,1.2} s:

(1) $$y_{\tau }\left(t\right)=I\left\{t_{\mathrm{ev}}\in \left(t,t+\tau \right]\right\}.$$

A positive label indicates that the proxy event will trigger within the next τ seconds. We focus on τ=0.2 s as the primary operating point; larger horizons are reported mainly as sensitivity checks of the same calibration interface (Section 5). To preserve causal semantics, we use pre-event-only slicing for event runs: slices are generated only up to (but not including) $t_{\mathrm{ev}}$, and safe runs (no proxy trigger) contribute only negative slices. Supplementary Section S2 provides an explicit pre-event negative ablation, confirming that post-event negatives are absent by construction.

We intentionally use a non-overlapping default stride equal to the window length. This matches the intended 10 Hz operator interface, avoids inflating the effective sample count through heavily overlapping windows, and reduces adjacent-slice dependence relative to a 0.05 s stride. A smaller stride could improve temporal quantization of warning time, but it does not remove the requirement of a full 0.1 s past history, and it strengthens overlap-induced correlation. To make these design choices explicit rather than heuristic, Supplementary Section S10 reports protocol-level design ablations for stride, window length, and run-level aggregation (Supplementary Tables S11 and S14).

### 3.4. Proxy Trigger Time $t_{ev}$: Parameter-Aware Route-B Predicate Family

We define $t_{\mathrm{ev}}$ using a conservative proxy based on three physics-motivated stability-margin predicates (Route-B family): (i) a friction-limited lateral envelope, (ii) a yaw-consistency deviation scaled by friction and wheelbase, and (iii) a load-transfer pre-warning scaled by center-of-gravity height and track width. Each predicate depends on the friction coefficient μ and vehicle signature parameters (wheelbase *L*, track *t*, and center-of-gravity height *h*), making platform differences explicit on the label side. The proxy event time is defined as the earliest time any predicate is violated for K=2 consecutive frames (debouncing). In our corpus, yaw-consistency is the dominant earliest trigger, while envelope violations are rare (Supplementary Table S3); this reflects the operating regimes captured by the terminal-phase snippets.

We emphasize that these “unsafe” labels are proxy triggers for entry into severe loss-of-margin regimes, not accident reports. The contribution of the paper is, therefore, proxy-triggered warning calibration: we calibrate alarms for a conservative, safety-relevant surrogate event and do not claim to estimate true crash or rollover probabilities directly.

### 3.5. Signals, Vehicle/Environment Channels, and Slice Features

Each run provides time-varying onboard channels such as longitudinal/lateral velocity and acceleration ($v_{x},v_{y},a_{x},a_{y}$), yaw rate *r*, and (when available) attitude and sideslip-related signals. To capture platform and environment variability, we attach: (i) a vehicle signature (e.g., *L*, *t*, *h*, steering ratio) and (ii) an environment/initial-condition descriptor including tire–road friction μ and initial speed $v_{0}$. Across the corpus, we observe G=3 distinct vehicle platforms, reported as anonymized IDs (vehicle_1, vehicle_2, vehicle_3) via vehicle_id. The kinematic channels are logged at 100 Hz, while the signature and environment descriptors enter as run-level metadata (treated as constant within a run); in a deployed system, they can be supplied by configuration or by existing estimators if available.

Importantly, the proposed calibration interface does not rely on any specific metadata channel. If certain descriptors are unavailable online, the hazard scorer can be trained and calibrated on the remaining onboard channels, and the same conformal thresholding procedure applies to the resulting score stream. What matters statistically is that calibration negatives and deployment negatives remain matched in the final score distribution.

For each signal channel, we compute robust summary statistics over each 0.1 s slice, including mean, standard deviation, extrema, selected percentiles, and RMS. All features remain in physical units; missing values are median-imputed using statistics fitted on the training split and reused for calibration/test to ensure reproducibility. This is a reproducible baseline for isolated missing entries, not a claim that static imputation is the correct fail-safe response to sustained sensor dropout. If a critical channel fails for multiple consecutive updates in deployment, the relevant question becomes whether the negative score distribution shifts enough to alter false-alarm control. We therefore report dedicated missing-signal robustness results in Supplementary Section S11 together with the corresponding stress-test protocols (Supplementary Table S15), and recommend a degraded-mode or abstaining policy for prolonged dropouts.

The same logic applies to the friction descriptor μ. In this dataset, μ is available as scenario metadata and treated as constant within a run; in a deployed system, it may instead come from configuration, a road-class prior, or an upstream estimator with delay and noise, or be omitted from the feature set entirely. The conformal layer operates on the score stream produced by the chosen feature pipeline, but its realized FPR still depends on calibration/deployment alignment of that score distribution. We, therefore, report explicit run-level μ bias/noise/coarsening sensitivity together with a no-μ ablation in Supplementary Section S12. Because the corpus does not contain a time-varying within-run μ estimate, those experiments should not be interpreted as validation against within-run estimator lag.

### 3.6. Splits, Base Rates, and the Warnability Upper Bound

We partition the dataset into disjoint Train/Val/Cal/Test splits at the run level. The Train split fits the scorer, the Val split is reserved for model/hyperparameter tuning, the Cal split is used only to select conformal thresholds for a target risk α, and all performance is reported on the held-out Test split. Table 1 summarizes the dataset scale, and Table 2 reports test-set base rates for each horizon. Notably, the τ=0.2 s task is extremely imbalanced (positive fraction 0.64% on test), which is why AUPRC, reliability, and operating-point precision are emphasized alongside AUROC.

#### Warnability

Because slices are past-only and require a full 0.1 s history, some event runs are inherently unwarnable for the run-level monitor used in this paper. For a generic slice window *w*, stride *d*, and rolling-mean length *k*, the first eligible pre-event aggregated statistic cannot appear earlier than:

(2) $$t_{\mathrm{elig}}=w+\left(k-1\right)d.$$

A run is, therefore, warnable if and only if $t_{\mathrm{ev}}>t_{\mathrm{elig}}$. Under the default 0.1 s = 0.1 s = 0.1 s design and k=2, this reduces to $t_{\mathrm{ev}}>0.2$ s. To avoid misinterpreting episode-level performance under terminal-phase alignment, we therefore report the warnable rate and decompose the event warning rate (EWR) as follows:

(3) EWR=Pr(warnable)×Pr(alarm∣warnable).

Throughout the run-level analysis, “warnable” refers to this monitor-specific notion of warning feasibility. The decomposition separates a data-induced upper bound (availability of a pre-event aggregated statistic under the chosen monitor) from the conditional effectiveness of the alarm on warnable runs.

## 4. Methods

### 4.1. Pipeline Overview

**Figure 2:**
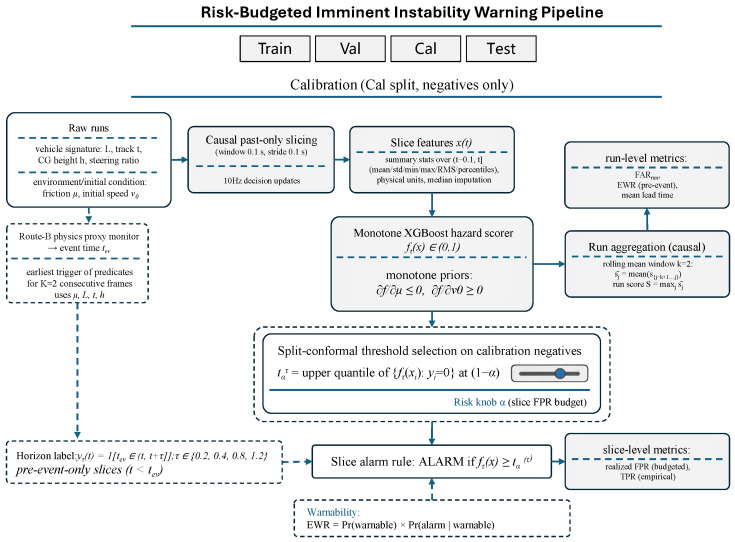
End-to-end pipeline. Past-only slicing (0.1 s window, 0.1 s stride) produces features and horizon-specific labels. A hazard scorer (optionally trained with monotonic constraints) outputs $f_{\tau }\left(x\right)\in \left[0,1\right]$. Split-conformal calibration on held-out negative slices selects thresholds that implement an operator-facing false-alarm budget α (globally or per vehicle).

Figure 2 summarizes the end-to-end pipeline. Given a run sampled at 100 Hz, we build causal, past-only slices of duration 0.1 s with a stride of 0.1 s. Each slice yields a feature vector x(t) and a horizon-specific label $y_{\tau }\left(t\right)$ for τ∈{0.2,0.4,0.8,1.2} (Equation (1)). For each horizon, a lightweight hazard scorer $f_{\tau }\left(x\right)\in \left(0,1\right)$ produces an imminent-instability score. We then select an alarm threshold from a disjoint calibration split using split-conformal calibration on negative slices. The resulting alarm rule is parameterized by a single operator-facing false-alarm budget α that targets the slice-level false positive rate (FPR). We refer to this global thresholding interface as Scheme A. To address fleet heterogeneity, Scheme B applies Mondrian (group-conditional) calibration with group map g(x)=vehicle_id, i.e., one threshold per vehicle with hierarchical back-off when support is low.

The default (w,d,k)=(0.1s,0.1s,2) design is intentional. It matches a 10 Hz decision interface, avoids additional overlap dependence between adjacent slices, and keeps the number of audited decisions aligned with the number of actual decision updates. Supplementary Table S11 states exactly which stages are rerun in the stride, window-length, and aggregation ablations. Those studies show a consistent trade-off: shorter windows or smaller *k* increase monitor-specific warnability, whereas larger windows or larger *k* suppress chatter but push the first eligible aggregated statistic later in time. We therefore treat (0.1s,0.1s,2) as an engineering default rather than a claim of global optimality.

### 4.2. Route-B Proxy and Event-Time Definition

We define a conservative proxy event time $t_{\mathrm{ev}}$ as the earliest time any of three stability-margin predicates is violated for K=2 consecutive frames (debouncing). The predicates are parameter-aware: they depend on tire–road friction μ and vehicle signature parameters (wheelbase *L*, track *t*, and center-of-gravity height *h*), which makes platform differences explicit on the supervision side.

#### 4.2.1. Friction-Limited Lateral Envelope

We flag lateral infeasibility when:

(4) $$|a_{y}|>\mu g.$$

#### 4.2.2. Yaw-Consistency

Let *r* denote yaw rate and δ denote the road-wheel steering angle (estimated from steering wheel angle via the steering ratio). Under a small-angle kinematic approximation, a nominal yaw rate is $r_{\mathrm{ref}}\left(v,\delta ,L\right)\approx \frac{v}{L}\;\delta$. Here *v* is forward speed in m/s, *L* is wheelbase in m, and *r* and δ are represented in a consistent logged angle convention. We trigger when the deviation exceeds a friction- and speed-scaled engineering bound:

(5) $$\left|r-r_{\mathrm{ref}}\left(v,\delta ,L\right)\right|>\gamma_{r}\;\frac{\mu g}{\max(v,v_{\min})},$$

The physically meaningful trend is carried by $\mu g/\max(v,v_{\min})$: for fixed μ, doubling speed halves the allowed yaw-rate mismatch, whereas for fixed speed, higher friction increases the tolerance proportionally. The low-speed floor $v_{\min}$ prevents the bound from diverging near standstill. The coefficient $\gamma_{r}$ absorbs the angle-unit convention and the desired tolerance scale, so it should be read as a frozen engineering threshold parameter rather than a universal physical constant. In all experiments, $v_{\min}=2.0\;m/s$ and $\gamma_{r}=31.232$ are tuned on the development split and then frozen before calibration/test evaluation. Table 3 reports the exact numeric constants used in the implementation.

#### 4.2.3. Load-Transfer Pre-Warning

We approximate the load-transfer ratio by geometry and lateral acceleration:

(6) $${\mathrm{LTR}}_{\mathrm{est}}\approx \frac{2h}{t}\;\frac{|a_{y}|}{g},\;\mathrm{trigger}\;\mathrm{if}\;{\mathrm{LTR}}_{\mathrm{est}}\geq \lambda_{\mathrm{LTR}},$$

with $\lambda_{\mathrm{LTR}}=0.8$. In the present corpus, this term is never the earliest trigger, but we retain it to preserve the composite safety envelope and to support datasets where rollover-like loading is more prominent.

**Figure 3:**
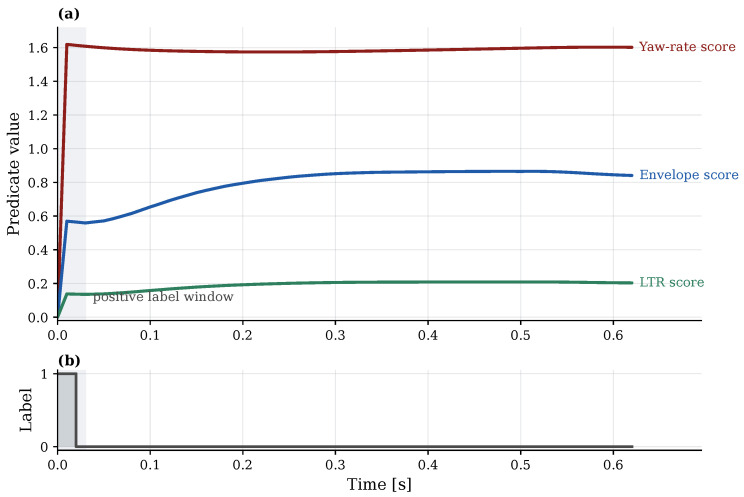
Labeling example (single run). (**a**) Example trajectories of the three proxy-component scores (yaw-rate, friction-limited envelope, and LTR) in a single run; the shaded region marks slice end times whose labels are positive for the illustrated horizon, i.e., those for which the proxy event time $t_{\mathrm{ev}}$ falls in (t,t+τ]. (**b**) Corresponding binary horizon label $y_{\tau }\left(t\right)=I\left\{t_{\mathrm{ev}}\in \left(t,t+\tau \right]\right\}$ for the same run.

Figure 3 illustrates the resulting event time and horizon labels on an example run.

### 4.3. Implementation Details and Fixed Constants

To improve reproducibility, Table 3 consolidates the fixed constants, tuning parameters, and implementation fields used throughout the study.

### 4.4. Hazard Scorer from Past-Only Slices

For each horizon τ, we train a gradient-boosted decision tree (GBDT) model (XGBoost) [36] to map slice features to a hazard score:

(7) $$f_{\tau }\left(x\right)=\sigma \left(\sum_{m=1}^{M}T_{m}\left(x\right)\right),\;\sigma \left(z\right)=\frac{1}{1+e^{-z}},$$

where $T_{m}$ are regression trees and σ is the logistic link. Class imbalance is handled using standard reweighting (e.g., scale_pos_weight based on the Train split).

#### 4.4.1. Physics-Consistent Monotonicity

When the environment/initial-condition descriptors $\left(\mu ,v_{0}\right)$ are available (as scenario metadata or online estimates), we optionally encode two weak physical priors as global monotone constraints during training:

(8) $$\frac{\partial f_{\tau }}{\partial \mu }\leq 0,\;\frac{\partial f_{\tau }}{\partial v_{0}}\geq 0,$$

i.e., predicted risk is non-increasing in friction and non-decreasing in initial speed. These constraints are not required for the conformal interface; they serve as a mild regularizer that improves interpretability and encourages nested acceptance behavior as surfaces become more slippery or initial speeds increase.

#### 4.4.2. Feature-Set Flexibility and Deployment-Side Metadata

The scorer may include or exclude μ and other metadata. Split-conformal calibration does not require those specific variables, but it does require the negative score distribution seen at calibration to remain representative of deployment. Thus, uncertainty in μ estimation or missingness in critical channels matters through any induced shift in the score stream that ultimately reaches the calibration layer.

### 4.5. Distribution-Free Threshold Calibration (Slice Level)

Let $C^{\left(\tau \right)}={\left\{\left(x_{i},y_{i}\right)\right\}}_{i=1}^{n}$ denote the calibration set for horizon τ and let $C_{0}^{\left(\tau \right)}=\left\{i:y_{i}=0\right\}$ denote calibration negatives (non-imminent slices). Write $s_{i}=f_{\tau }\left(x_{i}\right)$ for $i\in C_{0}^{\left(\tau \right)}$. For a multiset of scores $\left\{s_{i}\right\}$ and q∈(0,1), define the (conservative) upper empirical quantile:

(9) $${\mathrm{Quantile}}_{q}^{↑}\left(\left\{s_{i}\right\}\right)≜\min\left\{t:\#\left\{i:s_{i}\leq t\right\}\geq \left\lceil q\;\right|\left\{s_{i}\right\}\left|\right\rceil \right\}.$$

The global split-conformal threshold at risk level α is:

(10) $$t_{\alpha }^{\left(\tau \right)}={\mathrm{Quantile}}_{1-\alpha }^{↑}\left(\left\{s_{i}:i\in C_{0}^{\left(\tau \right)}\right\}\right).$$

At deployment time (or in evaluation), we alarm on a slice if $f_{\tau }\left(x\right)>t_{\alpha }^{\left(\tau \right)}$ and accept otherwise. We write the alarm rule in strict form so the one-sided conformal statement does not rely on a no-ties assumption; an implementation that prefers $f_{\tau }\left(x\right)\geq t_{\alpha }^{\left(\tau \right)}$ should resolve threshold ties explicitly (e.g., by infinitesimal score jitter or randomized tie-breaking). Under exchangeability between calibration and deployment negatives, this construction provides finite-sample, one-sided control of the marginal slice-level FPR on negative slices up to a small discretization slack [1,2].

### 4.6. Vehicle-Conditioned Mondrian Calibration (Scheme B)

To account for cross-vehicle heterogeneity, we use Mondrian (group-conditional) calibration with a group map:

(11) g(x)=vehicle_id∈G.

For each vehicle group g∈G with calibration negatives $C_{0,g}^{\left(\tau \right)}$, we compute a group threshold:

(12) $$t_{\alpha }^{\left(\tau \right)}\left(g\right)={\mathrm{Quantile}}_{1-\alpha }^{↑}\left(\left\{f_{\tau }\left(x_{i}\right):i\in C_{0,g}^{\left(\tau \right)}\right\}\right).$$

If a vehicle has insufficient calibration support, we back off to the global threshold, which preserves the corresponding coarser conformal validity. This yields a practical fleet interface: one shared scorer $f_{\tau }$ and vehicle-specific thresholds that implement the same risk knob α within each vehicle. We emphasize that Scheme B is a budgeting and auditing interface, not a claim of identical realized per-vehicle error rates under finite support.

### 4.7. Run-Level Deployment View: Aggregation and Warnability Decomposition

Slice-level FPR control is the primary guarantee. For deployment interpretation, we additionally report run-level behavior using a causal aggregation of slice scores. Let a run yield a sequence of slice scores ${\left\{s_{j}\right\}}_{j=1}^{J}$ at 10 Hz. We compute a rolling-mean statistic of window length *k*:

(13) $${\bar{s}}_{j}=\frac{1}{k}\sum_{\ell =j-k+1}^{j}s_{\ell },\;j\geq k,$$

and define the run score as $S=\max_{j\geq k}{\bar{s}}_{j}$. The first eligible aggregated statistic appears no earlier than $t_{\mathrm{elig}}=w+\left(k-1\right)d$, where *w* is slice length, and *d* is slice stride; equivalently, a run is warnable if and only if $t_{\mathrm{ev}}>t_{\mathrm{elig}}$ (Equation (2)). We calibrate a run-level threshold $T_{\alpha_{\mathrm{run}}}$ on safe runs (runs with no proxy trigger) to target a safe-run false-alarm budget $\alpha_{\mathrm{run}}$ and use the same strict-exceedance convention as at slice level. We then report: (i) FAR_run_, the fraction of safe runs with $S>T_{\alpha_{\mathrm{run}}}$, and (ii) EWR, the fraction of event runs that produce at least one alarm prior to $t_{\mathrm{ev}}$. For additional interpretability, we also report FAR_time_: the fraction of eligible time indices in safe runs for which the rolling-mean statistic exceeds the run threshold, i.e., ${\bar{s}}_{j}>T_{\alpha_{\mathrm{run}}}$.

Finally, to avoid conflating algorithmic effectiveness with data-imposed limits, we decompose EWR as in Equation (3). For the run-level monitor in Equation (13), a run is warnable if it contains at least one pre-event aggregated statistic ${\bar{s}}_{j}$, equivalently at least *k* causal pre-event slice scores. We, therefore, report both the warnable rate Pr(warnable) and the conditional effectiveness Pr(alarm∣warnable). For an early-warned event run, let $t_{\mathrm{first}\;\mathrm{alarm}}$ denote the first pre-event time at which ${\bar{s}}_{j}>T_{\alpha_{\mathrm{run}}}$. We define lead time as follows:

(14) $$\mathrm{Lead}=t_{\mathrm{ev}}-t_{\mathrm{first}\;\mathrm{alarm}},$$

and average it over warned event runs. Because the empirical run-level monitor can cross its threshold before the first slice whose label satisfies $y_{\tau }\left(t\right)=1$, lead time can exceed the labeling horizon τ.

### 4.8. Runtime/Resource Profiling Protocol

Because the interface targets 10 Hz updates, we report a host-side batch-1 runtime/resource profile of the Python prototype on the measured desktop host. The benchmark host is an Intel^®^ Core™ i7-14700KF CPU (MaxClockSpeed 3.40 GHz; 20 physical cores; 28 logical processors) with 95.84 GB RAM running Microsoft Windows 11 Pro (Version 10.0.26200) and Python 3.10.18. We instrument the deployed batch-1 pipeline to log per-update latency for (i) causal feature extraction, (ii) single-horizon model inference, (iii) threshold comparison/alarm logic, and (iv) the causal rolling-mean update, together with (v) serialized model footprint on disk and (vi) peak RAM. Here, peak RAM denotes the process-level peak resident memory observed during the benchmark rather than an additive per-component budget. The resulting numbers quantify host-side prototype timing relative to a 100 ms update cycle on that machine. Because library threading and hardware parallelism are host-dependent, they should be read as evidence of host-side prototype feasibility at 10 Hz rather than as a single-core benchmark or an embedded-ECU claim. In the Section 5 reports the resulting profile.

### 4.9. Metrics and Reporting Conventions

We report metrics in four layers. First, slice-level FPR@α on negative slices is the sole formal guarantee. Second, slice-level model diagnostics include AUPRC, AUROC, expected calibration error (ECE; 20 bins), and reliability curves. Third, run-level deployment metrics include FAR_run_, EWR, EWR on runs that are warnable under the chosen run-level monitor, and lead time. Fourth, assumption diagnostics include a run-blocked bootstrap and intentional subdomain-mismatch stress tests. Table 4 summarizes this mapping.

The conformal guarantee is one-sided and marginal: it targets false alarms on exchangeable negatives. Temporal dependence, sensor missingness, estimator lag, or covariate shift matter only insofar as they alter the negative score distribution between calibration and deployment. We, therefore, report explicit assumption diagnostics in the main text and complementary robustness studies in the Supplementary Information.

## 5. Results

Unless stated otherwise, all results are reported on the held-out test split. We emphasize the primary operating point τ=0.2 s and treat larger horizons as sensitivity checks of the same calibration interface.

### 5.1. Metric Hierarchy: What Each Result Answers

Before presenting numbers, we state explicitly what each metric is meant to answer. Table 4 separates the sole formal guarantee (slice-level FPR@α on negatives) from slice-level model diagnostics (AUPRC, AUROC, ECE, and reliability), run-level deployment KPIs (FAR_run_, EWR on warnable runs, and lead time), and assumption/resource audits. This hierarchy is important because a reader should not have to infer which metric corresponds to which deployment question.

### 5.2. Dataset Scale, Base Rates, and Terminal-Phase Alignment

Table 1 summarizes run and slice counts across Train/Val/Cal/Test, while Table 2 reports the test-set base rates for each horizon. The primary task at τ=0.2 s is highly imbalanced, with only 0.638% positives on test.

Terminal-phase alignment is a defining property of the corpus. Figure 1 shows that the proxy trigger time $t_{\mathrm{ev}}$ is heavily concentrated near the start of each run, which limits the time-to-event support available for causal pre-warning. This motivates treating the problem as proxy-triggered imminent recognition rather than multi-second forecasting.

### 5.3. Slice-Level Fidelity: AUPRC, Calibration, and Operating-Point View

**Figure 4:**
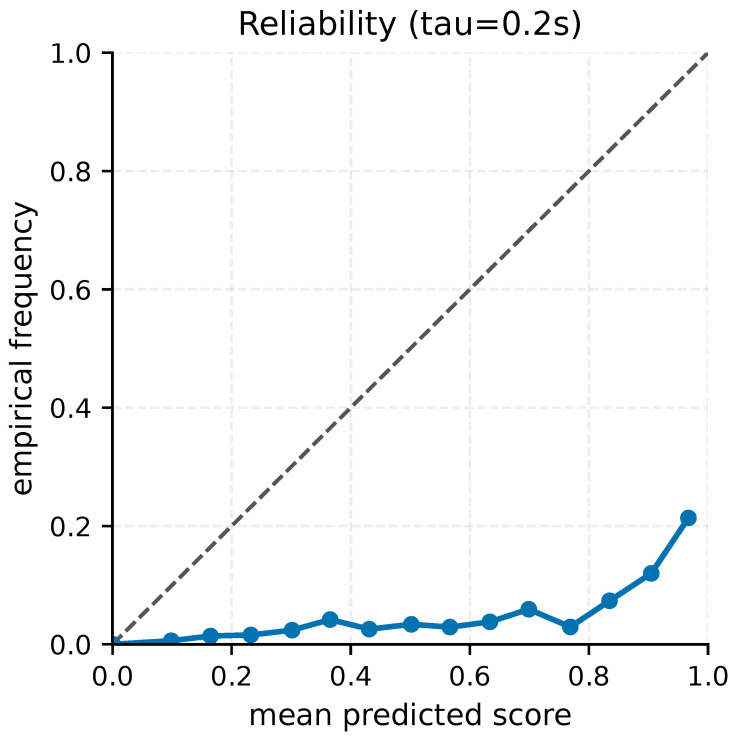
Primary-horizon reliability curve. Reliability diagram on the held-out test split at τ=0.2 s. The dashed diagonal denotes the ideal-calibration reference y=x; The curve complements AUPRC and AUROC by showing how predicted risk aligns with empirical event frequency in the rare-event regime.

Table 5 reports slice-level model diagnostics. We place AUPRC before AUROC because AUROC can appear overly optimistic under extreme class imbalance. At τ=0.2 s, the scorer attains AUPRC 0.251, which corresponds to roughly 39× lift over the base rate, while ECE remains small (0.034). Figure 4 shows the reliability curve at the primary horizon.

AUPRC and reliability are not the whole deployment story: once the monitor is calibrated to a particular false-alarm budget, practitioners also care about operating-point precision (PPV) and alarm prevalence. Table 6, therefore, reports slice-level PPV at the deployed conformal thresholds. At the primary horizon, PPV is 23.4% at α=1% and 10.8% at α=5% for the global threshold, while the vehicle-conditioned Mondrian threshold yields a very similar 10.7% PPV at α=5%. These values are modest in absolute terms, but that is expected under a 0.638% base rate and is precisely why a deployment paper should report precision explicitly rather than relying on AUROC alone.

### 5.4. Distribution-Free False-Alarm Control on Negative Slices

We next evaluate the calibrated operating points produced by split-conformal thresholding on calibration negatives. At the primary horizon, global calibration yields realized FPR close to the prescribed budgets while preserving high slice-level TPR on imminent positives at α=5% (Table 7). The guarantee here is one-sided and marginal: the calibrated threshold controls false alarms on negative slices under exchangeability, while TPR is an empirical availability statistic.

#### Vehicle-Conditioned Risk Budgeting (Scheme B)

To address cross-vehicle heterogeneity, we apply vehicle-conditioned Mondrian calibration (Equation (12)), which assigns each vehicle its own threshold while retaining a single global knob α. Table 8 shows that vehicle-conditioned calibration achieves essentially the same aggregate slice-level operating point as the global threshold at τ=0.2 s (FPR≈0.053, TPR≈0.980). The value of Scheme B is therefore a per-vehicle budgeting and auditing interface rather than guaranteed equalization of realized per-vehicle rates; Supplementary Table S11 shows that finite-sample per-vehicle FPRs still fluctuate around the nominal budget.

### 5.5. Run-Level Deployment View, Warnability, and Uncertainty

While our formal statement is slice-level FPR control on negative slices, practitioners also reason at the run level: “does a safe run alarm at least once?” and “does an event run receive any pre-event warning?” We therefore report a run-level deployment view using the rolling-mean aggregation (Equation (13), k=2) and a safe-run risk knob $\alpha_{\mathrm{run}}$.

**Figure 5:**
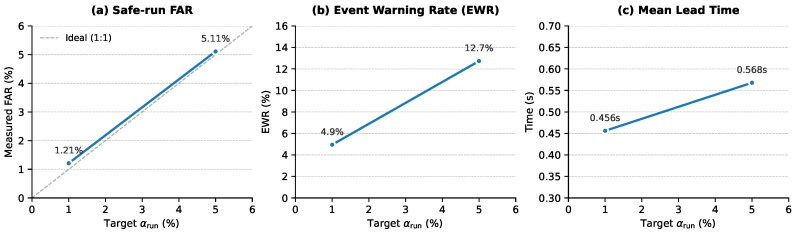
Run-level deployment view (global calibration; rolling-mean aggregation, k=2). Each point corresponds to a run-level risk budget $\alpha_{\mathrm{run}}$ calibrated on safe runs. We report FAR_run_ (safe runs with at least one alarm) and EWR (event runs with at least one pre-event alarm). Under terminal-phase alignment, overall EWR is upper-bounded by the warnable rate; Table 9 reports the corresponding decomposition.

Figure 5 illustrates the trade-off between safe-run false alarms and event warnings, and Table 9 reports the key run-level metrics at τ=0.2s.

At $\alpha_{\mathrm{run}}=5\%$, FAR_run_ concentrates near the budget. Because the corpus is terminal-phase aligned, overall EWR is limited by the warnable rate induced by causal slicing, early proxy triggers, and the requirement that the k=2 rolling-mean monitor observe at least one pre-event aggregated statistic. The decomposition in Table 9 shows that only about 18% of event runs are warnable under this monitor, but conditional effectiveness on warnable runs is high (0.697 at $\alpha_{\mathrm{run}}=5\%$), with a mean lead time of 0.568 s. Lead time is defined as in Equation (14), namely $t_{\mathrm{ev}}-t_{\mathrm{first}\;\mathrm{alarm}}$ on warned event runs. Because the empirical run-level alarm can cross threshold before the first τ-positive slice, observed lead times can exceed τ; the reported 0.568 s mean is therefore an empirical anticipation margin rather than a contradiction of the τ=0.2 s label definition. In this dataset, EWR on warnable runs and lead time are therefore more informative deployment KPIs than overall EWR alone.

Table 10 reports bootstrap confidence intervals using the run—not the slice—as the resampling unit. At $\alpha_{\mathrm{run}}=5\%$, FAR_run_ has a 95% CI of [0.047, 0.056], EWR_w_ has a 95% CI of [0.658, 0.737], and mean lead time has a 95% CI of [0.528, 0.607] s.

### 5.6. Assumption Diagnostics and Guarantee Boundary

**Figure 6:**
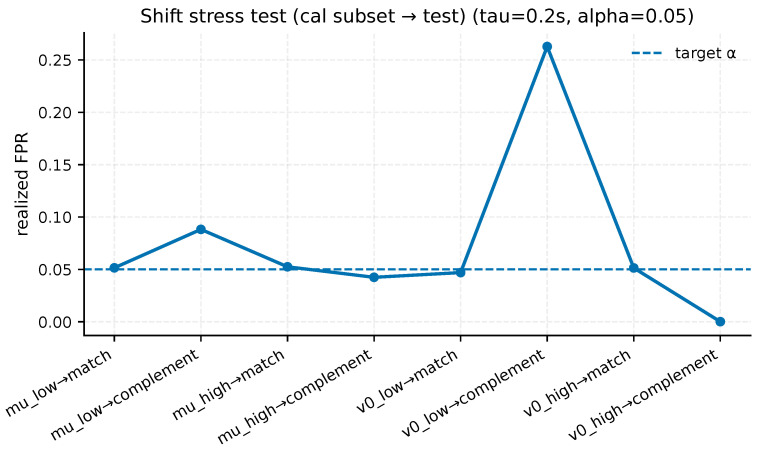
Intentional subdomain-mismatch stress test at τ=0.2 s and α=5%. When calibration and test negatives are intentionally mismatched across friction or initial-speed subdomains, realized FPR can drift far above the nominal target, illustrating the dependence of the guarantee on distributional alignment between calibration and deployment negatives.

A conformal validity claim is meaningful only when its assumptions are made explicit. Table 11, therefore, summarizes two diagnostics relevant to the guarantee boundary. First, a run-blocked bootstrap recalibrates the threshold using one negative slice per calibration run, reducing within-run dependence in the calibration support. Second, an intentional subdomain-mismatch stress test calibrates on restricted negative subsets and evaluates on mismatched test subsets, making the consequences of non-exchangeability concrete. The exact subset definitions and negative support counts used in that stress test are spelled out in Supplementary Table S13, and the resulting mismatch profiles at α=5% are visualized in Figure 6.

These results sharpen the interpretation of the conformal guarantee. Under the blocked-bootstrap diagnostic, mean test FPR is 0.044 and remains in the range 0.040–0.049 over 200 resamples at nominal α=5%, suggesting that moderate within-run dependence does not overturn the primary budget in this dataset. By contrast, deliberate subdomain mismatch can inflate FPR much more severely: calibrating on low-$v_{0}$ negatives and evaluating on the complementary higher-speed negatives yields FPR 0.263. In other words, the guarantee is conditional on calibration and deployment negatives being distributionally aligned.

### 5.7. Deployment Audits: Runtime, Stride, Missingness, and μ Robustness

We also audit computational footprint and robustness to cadence, missingness, and run-level μ perturbation. Because the available μ variable is run-level metadata, the sensitivity results in this subsection concern run-level descriptor perturbation and omission rather than within-run estimator lag. Supplementary Table S11 makes the ablation protocol explicit: stride and window studies are rerun end-to-end on the same run-level split, whereas the *k* study reuses slice scores and recalibrates only the run-level threshold on safe runs.

Table 12 reports a host-side batch-1 runtime/resource profile of the Python prototype on the measured desktop host (Intel^®^ Core™ i7-14700KF, 20 cores/28 logical processors, 95.84 GB RAM, Windows 11 Pro 10.0.26200, Python 3.10.18). Feature extraction and single-horizon inference require 5.64 ms and 4.21 ms on average, respectively, yielding an end-to-end latency of 9.98 ms per update with a P95 of 10.64 ms. Peak process memory observed during the benchmark is about 125 MB (a process-level peak rather than a per-component additive budget), and the serialized scorer-plus-threshold footprint is 3.27 MB on disk. Relative to a 100 ms 10 Hz cycle, these measurements support host-side prototype feasibility at 10 Hz on the benchmark desktop host. They do not isolate single-core execution, and they do not establish feasibility on production embedded automotive hardware.

Supplementary Table S14 compares the default 0.10 s stride with a 0.05 s overlapping stride at fixed w=0.10 s and k=2. Reducing the stride raises EWR_w_ from 0.697 to 0.806, but it nearly doubles the number of evaluated slices (1.95× workload), slightly increases FAR_run_ (0.051→0.053), and does not improve mean lead time (0.568 s → 0.534 s). We therefore retain the 0.1/0.1 cadence as the default operator interface and interpret 0.05 s as an optional higher-cadence mode that changes decision frequency rather than per-update latency.

Supplementary Table S14 also shows why the default history window remains w=0.10 s. Shortening the window to 0.05 s advances the first eligible aggregated statistic from 0.20 s to 0.15 s and raises the warnable rate from 0.183 to 0.259, which lifts overall EWR from 0.127 to 0.154. However, slice FPR and FAR_run_ also rise slightly (0.055 and 0.054), while the mean lead time falls to 0.503 s. Enlarging the window to 0.20 s slightly lowers slice FPR to 0.049 and raises mean lead time to 0.613 s, but the first eligible aggregated statistic shifts to 0.30 s, warnability drops to 0.141, and overall EWR falls to 0.100. The default w=0.10 s is therefore an engineering balance between per-slice fidelity and monitor-specific warning feasibility.

Supplementary Table S14 also shows why the run-level monitor uses k=2. With k=1, warnability rises to 0.308 and overall EWR to 0.173, but false-alarm runs become substantially chattier, with 2.34 alarm onsets per false-alarm run. With k=3, chatter falls to 1.18, and FAR_run_ drops to 0.048, but warnability falls to 0.141 and overall EWR to 0.100. The default k=2 therefore trades a moderate FAR_run_ (0.051) for substantially less chatter than k=1 while preserving more warning feasibility than k=3.

Supplementary Table S15 addresses deployment-time missingness under explicitly defined stress scenarios: 10% independent yaw-rate masking, contiguous burst outages on $a_{y}$ or jointly on $v_{x}/a_{y}/r$, causal forward-fill with median fallback, and an abstaining degraded mode. Isolated 10% random yaw-rate dropout leaves the operating point essentially unchanged, but sustained burst dropout on $a_{y}$ or jointly on $v_{x}/a_{y}/r$ reduces EWR_w_ to the 0.585–0.620 range. A degraded abstaining fallback sharply lowers FPR to 0.022 under the joint-burst scenario, but at the cost of coverage dropping to 0.523 and EWR_w_ dropping to 0.333. This supports the practical recommendation that prolonged multi-channel outages should trigger degraded mode rather than continued reliance on static imputation alone.

Supplementary Table S16 summarizes sensitivity to run-level μ perturbation and a no-μ ablation. In the present dataset, μ is stored as run-level metadata, so we study bias, noise, coarsening, and omission rather than a time-varying within-run estimator. Moderate noise leaves the operating point close to baseline (e.g., σ=0.05 yields FPR 0.054 and EWR_w_ 0.734), while the retrained no-μ model remains very close to the baseline operating point (FPR 0.052, FAR_run_ 0.051, EWR_w_ 0.684). These results support a narrower conclusion: on this dataset, the warning interface is reasonably robust to moderate run-level μ descriptor perturbation and to omission of μ as a feature. They do not constitute an estimator-in-the-loop validation against within-run friction-estimator lag.

### 5.8. Secondary Analyses Retained in the Supplementary Information

The Supplementary Information retains the pre-event-only negative ablation, cross-horizon reliability curves, per-vehicle dispersion tables, group-granularity ablations, the physics reference monitor, and the optional path-margin diagnostic. These are useful secondary audit artifacts that complement the core story: proxy-triggered warning calibration, metric hierarchy, run-level warnability, and explicit assumption diagnostics.

## 6. Discussion

This paper studies calibrated warning for a conservative, safety-relevant surrogate event built from interpretable stability-margin predicates. The proposed monitor is not presented as a direct predictor of crashes or true instability outcomes, and the reported μ robustness analysis concerns run-level descriptor perturbation and omission rather than a within-run friction estimator with temporal lag.

What is guaranteed, and what is not.The only formal guarantee is slice-level FPR control on exchangeable negative slices. Everything else—AUPRC, TPR, operating-point precision, FAR_run_, EWR, and lead time—is empirical. Making this hierarchy explicit prevents readers from conflating statistical validity with downstream usefulness. The run-blocked bootstrap and subdomain-mismatch diagnostics reinforce this point: in the present dataset, moderate dependence does not overturn the nominal budget, but mismatched calibration and deployment subdomains can degrade realized FPR rapidly.

Why the proxy framing is important.The proxy event is conservative by design. It is intended to identify entry into severe loss-of-margin regimes that are relevant for safety intervention, not to claim that a crash or rollover has occurred. Framing the work this way keeps the contribution precisely scoped: the paper proposes an auditable warning interface for a safety-relevant surrogate, and the proxy itself remains open to refinement in future datasets with richer measurements or outcome annotations.

Run-level deployment interpretation under terminal-phase data.Because the corpus is terminal-phase aligned, overall episode-level warning rates are structurally capped by warnability. In this setting, EWR on warnable runs and lead time are the most informative deployment metrics, while overall EWR must be interpreted as a product of data availability and alarm effectiveness. This decomposition makes clear that the monitor should not be penalized for event runs that contain too few causal pre-event slices to form any pre-event aggregated statistic under the chosen run-level monitor.

Default 0.1/0.1 slicing, 0.1 s history, and k=2 aggregation.The design ablations show that (w,d,k)=(0.1s,0.1s,2) is a trade-off, not a magic setting. Reducing the stride to 0.05 s is a genuine cadence trade-off rather than a uniformly better choice: EWR_w_ rises from 0.697 to 0.806, but the number of evaluated slices almost doubles (1.95× workload), FAR_run_ increases slightly (0.051→0.053), and mean lead time does not improve (0.568 s → 0.534 s). Shortening the history window to 0.05 s increases warnability and overall EWR, but it slightly worsens slice/run-level false alarms and reduces mean lead time; enlarging the window to 0.20 s has the opposite effect and loses too many warning-feasible runs. Likewise, k=1 maximizes warnability but produces a noticeably chattier interface, while k=3 suppresses chatter at the cost of additional warning infeasibility. We therefore keep (0.1s,0.1s,2) as the main deployment interface and present the alternatives as explicit operating-mode trade-offs.

Runtime and deployment realism. On the measured desktop host (Intel^®^ Core™ i7-14700KF @ 3.40 GHz; 20 cores/28 logical processors; 95.84 GB RAM; Windows 11 Pro 10.0.26200; Python 3.10.18), the present Python prototype requires 9.98 ms per update on average (P95 10.64 ms), with a benchmark peak process memory of about 125 MB and a serialized model-plus-threshold footprint of 3.27 MB. The RAM number is a process-level peak rather than an additive per-component budget. Relative to a 100 ms 10 Hz cycle, these measurements support host-side prototype feasibility at 10 Hz on the benchmark desktop host. They should not be read as validation on production embedded automotive ECUs.

Missingness, friction estimation, and drift. Median imputation is a reproducible baseline, not a final degraded-mode strategy. The robustness experiments support that interpretation. Isolated random dropout on yaw rate leaves the operating point almost unchanged, but burst dropout on $a_{y}$ and especially joint burst dropout on $v_{x}/a_{y}/r$ cause a clear loss in warning effectiveness. A simple forward-fill policy partially recovers performance relative to static imputation, while a degraded abstaining policy reduces FPR sharply but pays for that with reduced coverage and EWR_w_. In other words, the correct deployment response to sustained outages is fault detection and degraded operation, not continued reliance on static imputation alone.

The same principle applies to the friction descriptor μ. In the present dataset, μ is run-level metadata, so the studied perturbations are bias, noise, coarsening, and omission rather than within-run temporal lag. Under those perturbations, the operating point remains fairly stable, and the no-μ retrained model stays close to baseline. The asymmetric response to positive versus negative bias is consistent with tree ensembles: only perturbations that cross learned split thresholds materially alter the score path. The resulting deployment interpretation is narrower: the present study supports robustness to moderate run-level μ descriptor error and shows that omission of μ is a viable fallback when online friction estimates are unavailable or fail confidence checks. It does not validate a time-varying friction-estimator pipeline with a within-run lag.

Taken together, the stress tests suggest a simple three-mode deployment policy. In nominal mode, the full scorer operates when the critical kinematic channels and run-level descriptors are available. If μ is unavailable or fails confidence checks, the system can fallback to the no-μ scorer calibrated on that feature set. If the joint observability of $\left\{v_{x},a_{y},r\right\}$ is lost for a sustained outage, the monitor should switch to a degraded or abstaining mode rather than continue to trust static imputation alone.

Fleet heterogeneity and practical recommendations. Vehicle-conditioned Mondrian calibration remains a useful deployment primitive, but its value should be described carefully. In the present corpus, it preserves nearly the same aggregate slice-level operating point as the global threshold while exposing a per-vehicle budgeting interface. Per-vehicle rates do not uniformly improve in finite samples, so their main value here is auditing and budgeting rather than guaranteed per-vehicle equalization. The main engineering recommendation is therefore to use the coarsest grouping that captures dominant heterogeneity while maintaining adequate calibration support, together with periodic recalibration and score-distribution drift monitoring.

Future work. The next scientific step is to validate the proxy-triggered interface in broader nominal-driving data and, ideally, in controller-in-the-loop studies where alarms gate or shape emergency maneuvers. The next engineering step is to repeat the runtime profile on representative embedded hardware and to evaluate truly time-varying friction estimation pipelines, because the current dataset only supports run-level μ perturbations rather than within-run lag analysis.

## 7. Conclusions

We studied last-moment vehicle-instability warning as a proxy-triggered imminent-recognition task: from a 0.1 s past-only slice, decide whether a conservative instability proxy will trigger within τ=0.2 s. A lightweight hazard scorer, combined with split-conformal calibration on negative slices, yields an auditable alarm rule with a slice-level false-alarm budget α and finite-sample, one-sided control of the negative-slice marginal slice-level FPR under exchangeability assumptions. Vehicle-conditioned (Mondrian) calibration further provides a per-vehicle budgeting interface without per-vehicle retraining.

Two points deserve emphasis. First, the contribution is a calibrated warning for a safety-relevant surrogate event rather than a direct prediction of true instability outcomes. Second, the evaluation is organized into a clear hierarchy: slice-level FPR@α is the formal guarantee, AUPRC/reliability diagnose rare-event model quality, run-level warnability-aware metrics provide the deployment view, and dependence/shift diagnostics expose where validity can degrade.

Beyond the core slice-level results, the paper also reports operating-point precision, run-level bootstrap uncertainty, a measured host-side runtime profile on a benchmark desktop host, stride-ablation trade-offs, missing-signal robustness, and sensitivity to run-level μ perturbation together with a no-μ fallback analysis. Together, these results show where the interface works, how it degrades under overlap, dropout, and distribution mismatch, and what remains to be validated on broader data and representative embedded hardware.

## Figures and Tables

**Table 1:** Dataset scale and split summary (runs and 0.1 s slices).

Split	Runs	Event Runs	Safe Runs	Slices
cal	8667	2309	6358	131,146
test	11,583	3071	8512	175,985
train	28,917	7672	21,245	438,141
val	8667	2292	6375	131,638

**Table 2:** Test-set base rates (positive fraction) for each alarm window τ. Positives indicate $t_{\mathrm{ev}}\in \left(t,t+\tau \right]$.

τ (s)	Test Slices	Pos Slices	Neg Slices	Pos Frac (%)
0.2	175,985	1122	174,863	0.638
0.4	175,985	2244	173,741	1.275
0.8	175,985	3962	172,023	2.251
1.2	175,985	5125	170,860	2.912

**Table 3:** Implementation constants and fixed design choices used in the pipeline.

Item	Value	Notes
Slice window length	0.1 s	Past-only slice duration used for all horizons.
Default slice stride	0.1 s	Non-overlapping default; the 0.05 s overlap ablation is reported in Supplementary Section S10.
Alarm horizons τ	{0.2, 0.4, 0.8, 1.2} s	Primary operating point is τ=0.2 s.
Rolling-mean window *k*	2	Run-level aggregation window.
Consecutive-frame debounce length *K*	2	Route-B proxy declares the earliest trigger only after K=2 consecutive frames.
LTR threshold $\lambda_{\mathrm{LTR}}$	0.8	Conservative load-transfer pre-warning threshold.
ECE bin count	20	Equal-width reliability bins.
Missing-value policy	train-median imputation	Fit on the Train split; deployment-side missingness is stressed explicitly in Supplementary Section S11.
Low-speed degeneracy threshold $v_{\min}$	2.0 m/s	Equation (5) low-speed safeguard.
Yaw-consistency scale $\gamma_{r}$	31.232	Development-set engineering scale in Equation (5); absorbs the logged angle-unit convention and tolerance level.
Mondrian low-support back-off threshold	300	Minimum per-group calibration support before global back-off.
XGBoost hyperparameters	max_depth = 6, lr = 0.05,	
n_estimators = 1200	subsample = 0.8, colsample = 0.9,	
reg_lambda = 1.0, early_stopping = 200.		
Monotonic constraints	$\mu \;:\;-1,\;v_{0}\;:\;+1$	Active feature count = 70; constraints are weak priors, not required for conformal validity.

**Table 4:** Metric hierarchy used in the manuscript. This table explicitly maps each metric to the deployment question it answers.

Metric Family	Representative Metric	Status	Deployment Interpretation
Formal guarantee	Slice-level FPR@α on negatives	Guaranteed (under exchangeability)	On truly negative slices, how often can the calibrated monitor false alarm?
Slice-level model quality	AUPRC, AUROC, ECE, reliability curve	Empirical diagnostic	How well separated and calibrated are the raw hazard scores before thresholding?
Run-level deployment KPI	FAR_run_, EWR_w_, lead time	Empirical KPI	How often does a safe run alarm, and how often is an event run warned when it is warning-feasible under the chosen run-level monitor?
Run-level context	Warnable rate, overall EWR	Empirical context	How much of the corpus is warning-feasible under terminal-phase alignment and the chosen run-level monitor?
Assumption audit	Blocked-bootstrap FPR, shift-stress FPR	Empirical diagnostic	How sensitive is FPR control to dependence and distribution mismatch?
Resource audit	Latency, model size, memory footprint	Engineering diagnostic	What host-side timing and memory headroom does the prototype leave at 10 Hz on the measured benchmark host?

**Table 5:** Slice-level predictive fidelity on the held-out test split. We foreground AUPRC because the task is strongly imbalanced; PR lift is the ratio between AUPRC and the test-set base rate. Higher is better for AUPRC/AUROC/PR lift; lower is better for ECE (20 bins).

τ (s)	Base Rate (%)	AUPRC	PR Lift	AUROC	ECE
0.2	0.638	0.251	39.34	0.986	0.034
0.4	1.275	0.284	22.27	0.977	0.057
0.8	2.251	0.424	18.84	0.979	0.057
1.2	2.912	0.526	18.06	0.981	0.052

**Table 6:** Operating-point precision (PPV) and alarm prevalence on the held-out test split at the primary horizon τ=0.2 s. Alarm prevalence is the fraction of test slices that alarm at the calibrated threshold.

α (%)	Scheme	Precision/PPV (%)	Alarm Prevalence (%)	$n_{\mathrm{alarm}}$
1	global	23.4	1.39	2453
5	global	10.8	5.81	10,222
5	vehicle-conditioned	10.7	5.86	10,319

**Table 7:** Global split-conformal thresholds and realized slice-level operating points are tested at the primary horizon τ=0.2 s.

α (%)	Threshold $t_{\alpha }^{\left(\tau \right)}$	FPR	TPR
1	0.963	0.011	0.511
5	0.124	0.052	0.982

The distribution-free guarantee is one-sided: it controls FPR on negative slices up to finite-sample slack; TPR is reported as availability only.

**Table 8:** Vehicle-conditioned (Mondrian) slice-level operating points on test at τ=0.2 s.

α (%)	FPR	TPR
1	0.011	0.532
5	0.053	0.98

Per-vehicle thresholds are computed on calibration negatives within each vehicle; small-support vehicles back off to the global threshold.

**Table 9:** Run-level deployment view (global calibration; rolling-mean aggregation with k=2). We report FAR_run_ under budget $\alpha_{\mathrm{run}}$, and decompose event warning rate (EWR) into the warnability upper bound and conditional effectiveness.

$\alpha_{\mathrm{run}}$ (%)	FAR_run_	FAR_time_ (%)	Warnable	EWR_w_	EWR	Lead (s)
1	0.012	0.10	0.183	0.271	0.049	0.456
5	0.051	0.42	0.183	0.697	0.127	0.568

Warnable rate is the fraction of event runs that contain at least one pre-event aggregated statistic under the k=2 rolling-mean monitor, equivalently at least two causal pre-event slices. EWR decomposes as $\mathrm{EWR}=\mathrm{Warnable}\times {\mathrm{EWR}}_{w}$. Lead time is $t_{\mathrm{ev}}-t_{\mathrm{first}\;\mathrm{alarm}}$ on warned event runs and may exceed τ because the empirical run-level alarm can cross threshold before the first τ-positive slice.

**Table 10:** Run-level uncertainty bands obtained by bootstrap resampling at the run level (1000 replicates). Lead time is measured as $t_{\mathrm{ev}}-t_{\mathrm{first}\;\mathrm{alarm}}$ on warned event runs.

Metric	Point Estimate	95% CI	Bootstrap Unit
FAR_run_ @ $\alpha_{\mathrm{run}}=5\%$	0.051	[0.047, 0.056]	safe run
EWR_w_ @ $\alpha_{\mathrm{run}}=5\%$	0.697	[0.658, 0.737]	event run
Overall EWR @ $\alpha_{\mathrm{run}}=5\%$	0.127	[0.116, 0.140]	event run
Lead time (s) @ $\alpha_{\mathrm{run}}=5\%$	0.568	[0.528, 0.607]	warned event run

**Table 11:** Assumption diagnostics at the primary horizon τ=0.2 s, illustrating sensitivity to dependence and deliberate distribution mismatch.

Diagnostic	Nominal Budget	Realized FPR	Interpretation
Run-blocked bootstrap calibration (200 replicates; one negative slice per calibration run)	α=5%	mean 0.044, range 0.040–0.049	Dependence-aware recalibration keeps FPR near target.
Friction mismatch stress (calibrate on $\mu_{\mathrm{low}}$, test on complementary subset)	α=5%	0.088	Mild mismatch already inflates FPR beyond target.
Initial-speed mismatch stress (calibrate on $v_{0,\mathrm{low}}$, test on complementary subset)	α=5%	0.263	Severe mismatch can sharply violate the target budget.

**Table 12:** Host-side batch-1 runtime and resource profile of the Python prototype on the measured desktop host. Peak RAM denotes the process-level peak resident memory observed during the benchmark and is therefore not additive across rows. Disk size denotes the serialized scorer-plus-threshold footprint on disk. The values support host-side prototype feasibility at 10 Hz on this host, but they do not establish feasibility on a production embedded automotive ECU.

Component	Mean (ms)	P95 (ms)	Peak RAM (MB)	Disk Size (MB)	Notes
Feature extraction (0.1 s slice)	5.64	5.86	125.36	–	window summarization
Single-horizon GBDT inference	4.21	4.75	125.36	–	batch = 1; 70 features
Threshold compare + alarm logic	<0.01	<0.01	125.36	–	scalar compare
Run-level rolling mean	0.04	0.04	125.36	–	k=2
End-to-end per-update latency	9.98	10.64	125.36	–	feature extraction + inference + alarm update
Serialized scorer + thresholds	–	–	–	3.27	on disk

## Data Availability

The benchmark provider/program name is anonymized in the manuscript. The raw runs used in this study are available on request from the corresponding author.

## Supplementary Materials

The following supporting information can be downloaded at: https://www.mdpi.com/article/10.3390/s26082302/s1, Figure S1: Histogram of $t_{\mathrm{ev}}$ from episode start (terminal-phase alignment); Figure S2: Pre-event negative ablation at τ=0.2 s: (a) realized pre-event FPR versus α and (b) calibrated thresholds; Figure S3: Vehicle-conditioned thresholds at τ=0.2 s and α=5% (Mondrian split-conformal); Figure S4: Reliability diagrams across horizons (test split); Figure S5: Score histograms across horizons (Train/Val/Cal/Test); Figure S6: Lead-time distribution for early-warned event runs at τ=0.2 s under a representative run-level budget $\alpha_{\mathrm{run}}=5\%$ (rolling-mean aggregation, k=2); Figure S7: Learned hazard scorer versus physics reference monitor (prule) at τ=0.2 s and α=5% (slice level) / $\alpha_{\mathrm{run}}=5\%$ (run level); Figure S8: Path-margin robustness at τ=0.2 s and α=5%: (a) certified-core coverage versus ϵ and (b) Monte Carlo decision flip rates under bounded perturbations; Figure S9: Realized test FPR under several threshold-selection baselines at τ=0.2 s; Figure S10: Per-vehicle FPR dispersion at τ=0.2 s under global versus vehicle-conditioned (Mondrian) calibration; Figure S11: Run-blocked bootstrap calibration at τ=0.2 s and α=5%; Figure S12: Subdomain mismatch stress test at τ=0.2 s; Figure S13: Stride ablation at the primary horizon; Figure S14: Missing-signal robustness at the primary horizon; Figure S15: Sensitivity to run-level μ perturbation and no-μ retraining at the primary horizon; Figure S16: Run-level bootstrap uncertainty bands; Table S1: Dataset provenance, access, and episode-curation summary for the benchmark used in this study; Table S2: Summary statistics of event times $t_{\mathrm{ev}}$ (seconds from episode start) for event runs; Table S3: Earliest-trigger predicate counts across all runs under the Route-B proxy event definition, where none denotes safe runs with no trigger; Table S4: Pre-event-only negative ablation at τ=0.2 s; Table S5: Vehicle-conditioned (Mondrian) slice-level operating points on the test split across alarm windows; Table S6: Run-level ablation at τ=0.2 s illustrating the effect of Mondrian group granularity and conservative across-μ smoothing (all at $\alpha_{\mathrm{run}}=5\%$ with rolling-mean aggregation k=2); Table S7: Physics reference monitor (prule) run-level performance at τ=0.2 s after run-level calibration ($\alpha_{\mathrm{run}}=5\%$, rolling-mean aggregation k=2); Table S8: Path-margin robustness at τ=0.2 s and α=5%; Table S9: Illustrative per-feature uncertainty budgets $u_{j}$ used for the path-margin robustness analysis at τ=0.2 s; Table S10: Threshold-selection baselines at τ=0.2 s; Table S11: Per-vehicle slice-level operating points at τ=0.2 s under global versus vehicle-conditioned (Mondrian) calibration, together with the protocol summary for the design ablations; Table S12: Run-blocked bootstrap calibration at τ=0.2 s and α=5%; Table S13: Subdomain mismatch stress tests at τ=0.2 s for α=1% and α=5%, together with the definitions of the restricted negative subsets used for intentional calibration–test mismatch; Table S14: Design ablations at the primary horizon τ=0.2 s; Table S15: Missing-signal robustness at τ=0.2 s and α=5% / $\alpha_{\mathrm{run}}=5\%$, together with the protocol definitions for the corresponding stress tests; Table S16: Sensitivity to run-level μ perturbation at the primary horizon τ=0.2 s; Table S17: Run-level bootstrap uncertainty bands (1000 replicates).
