# Supplementary material for: Vehicle-Conditional Split-Conformal Calibration for Risk-Budgeted Sub-Second Proxy-Triggered Vehicle Instability Warnings from Past-Only Sensor Slices
Source: Sensors (Basel). 2026 Apr 8;26(8):2302. doi: 10.3390/s26082302 (PMC13120485; doi:10.3390/s26082302)
Supplement: Supplementary file 1 [file sensors-26-02302-s001.zip › sensors-4165605-supplementary.pdf]

Supplementary Materials: Vehicle-Conditional Split-Conformal Calibration for Risk-Budgeted Sub-Second Proxy-Triggered Vehicle Instability Warnings from Past-Only Sensor Slices

Jinzhe Yang [1], Jianzheng Liu [1,*], Kai Tian [2], Yier Lin [1], and Junxia Zhang [1]

This Supplementary Information provides additional dataset diagnostics, ablations, robustness evidence, and proof sketches supplementing the main manuscript. It includes quantitative studies of stride cadence, missing-signal robustness, run-level μ-perturbation sensitivity, and run-level bootstrap uncertainty, together with supporting figures.

S1. Additional dataset diagnostics

Supplementary Table S1 summarizes dataset provenance and episode curation, including the run definition, metadata channels, and split rule used throughout the study.

Table S1. Dataset provenance, access, and episode-curation summary for the benchmark used in this study.

Item	Entry
Benchmark type	Anonymized controlled vehicle-dynamics benchmark corpus; each run is one terminal-phase single-maneuver episode rather than a long naturalistic driving segment.
Source statement	The provider/program name is anonymized in the manuscript; what is disclosed here is the acquisition/curation protocol relevant to validity. Each run contains 100 Hz onboard kinematic time series plus run-level vehicle/environment metadata.
Access statement	Raw runs are available on request from the corresponding author (see Data Availability statement).
Vehicle platforms	$G = 3$ anonymized platform IDs (`vehicle_1`, `vehicle_2`, `vehicle_3`).
Total scale	57,834 runs total: 15,344 event runs and 42,490 safe runs.
Run definition	A run is one maneuver instance; $t = 0$ is the episode start and t_{ev} is the first Route-B proxy trigger after $K = 2$ consecutive violating frames.
Episode alignment	Terminal-phase aligned: event episodes begin close to the onset of a critical handling regime, which concentrates proxy trigger times near run start (median 0.02 s; mean 0.197 s).
Safe-run definition	Safe runs are curated with the same episode-extraction logic but contain no Route-B proxy trigger over the recorded interval.
Onboard channels	100 Hz kinematic channels including v_x, v_y, a_x, a_y, yaw rate r, and attitude/sideslip-related signals when available.
Run-level metadata	Vehicle signature $(L, t, h,$ steering ratio$)$ and environment/initial-condition descriptors (μ, v_0); treated as constant within each run.
Episode extraction rule	Event runs are sliced causally only up to (but not including) t_{ev}; safe runs contribute only negative slices.
Split rule	Train/Val/Cal/Test splits are disjoint at the run level after episode curation, so all slices from a run remain in one split only.
Deployment interpretation	The corpus supports proxy-triggered terminal-phase warning evaluation; it does not directly validate naturalistic-driving crash or rollover outcomes.

Terminal-phase alignment. Figure S1 and Table S2 summarize the distribution of proxy event times t_{ev} (seconds from episode start) on event runs. As discussed in the main text, the corpus is curated as *terminal-phase* snippets extracted near the onset of a critical regime. Consequently, t_{ev} is strongly concentrated near the beginning of each run, which limits the time-to-event support available for causal, past-only pre-warning.

Which predicate triggers first? Table S3 reports which Route-B predicate is the earliest trigger in each run. Runs with `none` are safe runs (no trigger). In the present corpus, the

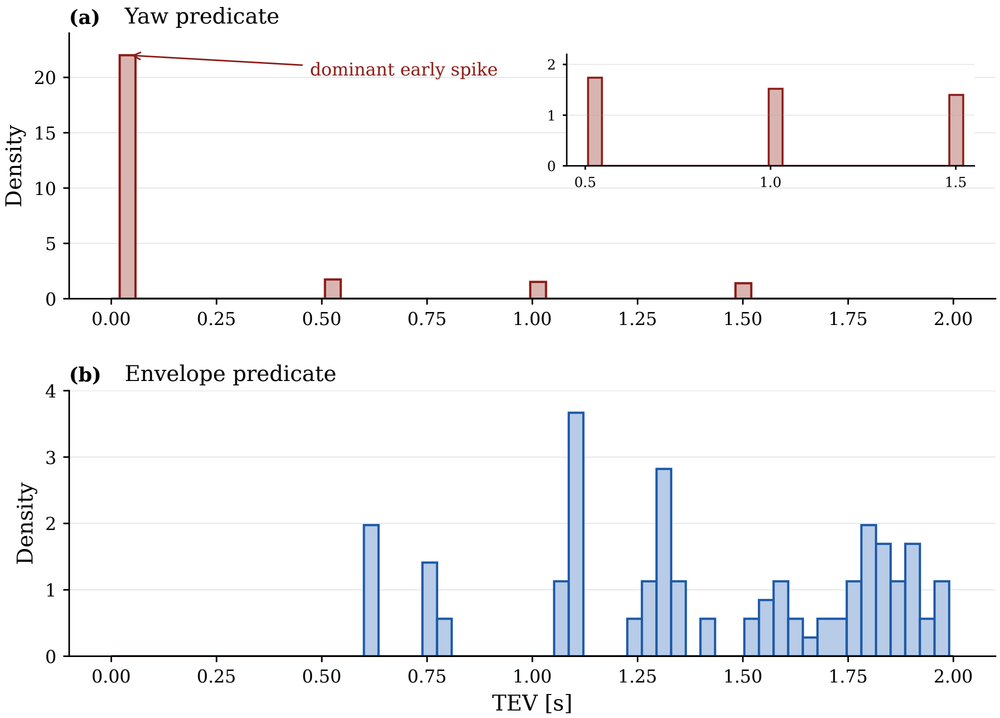

Figure S1. Histogram of t_{ev} from episode start (terminal-phase alignment).

Table S2. Summary statistics of event times t_{ev} (seconds from episode start) for event runs.

n event runs	mean	std	min	max	q01	q05	q10	q50	q90	q95	q99
15344	0.197	0.416	0.02	1.99	0.02	0.02	0.02	0.02	1.02	1.52	1.52

LTR pre-warning term is never the earliest trigger, but we retain it in the composite proxy so that the event definition continues to cover datasets where rollover-like loading is more prominent.

Table S3. Earliest-trigger predicate counts across all runs under the Route-B proxy event definition; `none` denotes safe runs with no trigger.

event_predicate	runs
none	42490
yaw	15242
envelope	102
LTR	0

S2. Pre-event-only negative semantics

A key requirement for deployability is that the false-alarm knob α corresponds to *pre-event* false alarms (warnings fired before the proxy event time). For event runs, we therefore define negative slices using only times strictly before the earliest trigger: $t < t_{ev}$. Figure S2 and Table S4 provide an explicit ablation comparing (i) calibration on all $y_\tau = 0$ slices ("mixed") and (ii) calibration on pre-event negatives only. Because our pipeline excludes post-event segments by construction, the post-event negative count is zero, and the calibrated thresholds/FPRs coincide.

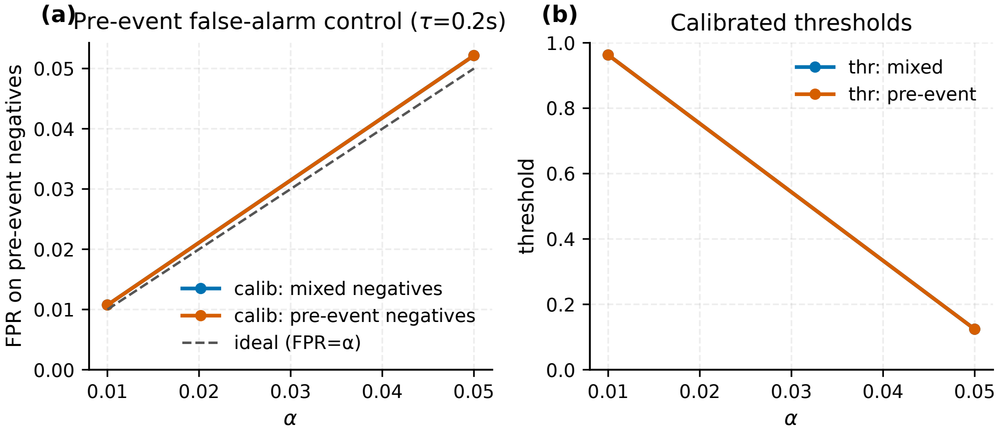

Figure S2. Pre-event negative ablation at $\tau = 0.2$ s: (a) realized pre-event FPR versus α and (b) calibrated thresholds.

Table S4. Pre-event-only negative ablation at $\tau = 0.2$ s. We compare calibration on all $y_\tau = 0$ slices ("mixed") versus calibration restricted to strictly pre-event slices ($t < t_{ev}$). In our pipeline, post-event negatives are absent by construction ($n^{post} = 0$), so thresholds and pre-event FPR coincide.

α (%)	t_α (mixed)	t_α (pre-event)	$n^{post}_{cal,0}$	$n^{post}_{test,0}$	FPR_{pre}	TPR
1	0.963	0.963	0	0	0.011	0.511
5	0.124	0.124	0	0	0.052	0.982

Calibration negatives: $n^{pre}_{cal,0} = 130{,}336$ and $n^{post}_{cal,0} = 0$; test negatives: $n^{pre}_{test,0} = 174{,}863$ and $n^{post}_{test,0} = 0$. The absence of post-event negatives verifies that the reported FPR control corresponds to *deployable pre-event* false alarms.

S3. Vehicle-conditioned Mondrian calibration (slice level)

Table S5 reports realized operating points induced by per-vehicle (Mondrian) split-conformal calibration across alarm windows. For each horizon, thresholds are computed on calibration negatives within each vehicle group (with hierarchical back-off to the global threshold when support is low). Figure S3 visualizes the vehicle-specific thresholds at $\tau = 0.2$ s under $\alpha = 5\%$, illustrating fleet heterogeneity and support-driven back-off.

Table S5. Vehicle-conditioned (Mondrian) slice-level operating points on the test split across alarm windows. Thresholds are computed per vehicle on calibration negatives and evaluated on test.

τ (s)	FPR@1%	TPR@1%	FPR@5%	TPR@5%
0.2	0.011	0.532	0.053	0.980
0.4	0.011	0.357	0.054	0.904
0.8	0.011	0.403	0.053	0.911
1.2	0.012	0.452	0.052	0.940

Per-vehicle thresholds implement a vehicle-conditional risk budget under the same knob α; vehicles with insufficient calibration support back off to the global threshold.

S4. Reliability and score distributions across horizons

We provide reliability diagrams and score histograms for all evaluated horizons $\tau \in \{0.2, 0.4, 0.8, 1.2\}$. These diagnostics help verify that the learned hazard scores remain well-behaved beyond the primary horizon.

S5. Group-granularity ablation (run level) and lead-time distribution

Although Scheme B uses vehicle-conditioned thresholds by default, one might consider finer Mondrian partitions (e.g., vehicle$\times\mu$-bin$\times v_0$-bin) to target more localized heterogene-

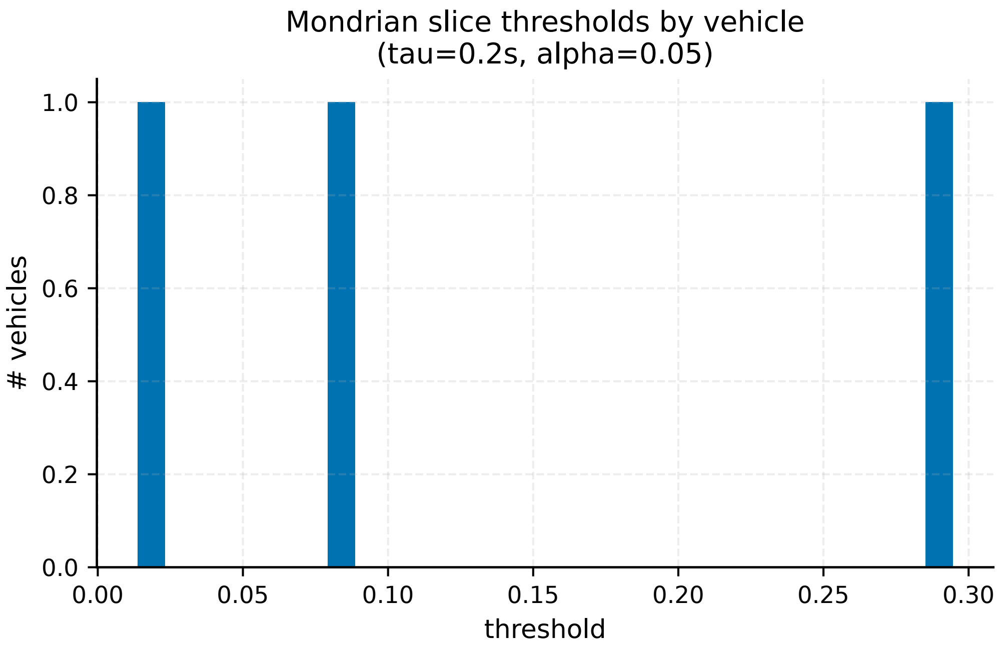

Figure S3. Vehicle-conditioned thresholds at $\tau = 0.2$ s and $\alpha = 5\%$ (Mondrian split-conformal). Cells with insufficient calibration support back off to the global threshold.

ity. Table S6 shows that overly fine partitions can become *overly conservative* under finite calibration support, substantially reducing run-level warning effectiveness (EWR), even when the same run-level false-alarm budget is used. This motivates using vehicle-only grouping as the default fleet heterogeneity primitive in the core evaluation. Throughout the run-level analysis, lead time denotes $t_{ev} - t_{first\,alarm}$ on warned event runs under the chosen rolling-mean monitor. Because the run-level alarm can cross the threshold before the first τ-positive slice, lead time may exceed τ.

Table S6. Run-level ablation at $\tau = 0.2$ s illustrating the effect of Mondrian group granularity and conservative across-μ smoothing (all at $\alpha_{run} = 5\%$ with rolling-mean aggregation $k = 2$).

Scheme	FAR_{run}	FAR_{time} (%)	EWR (all)	Mean lead (s)	Warnable rate	EWR (warnable)
global	0.051	0.42	0.127	0.568	0.183	0.697
vehicle$\times\mu \times v_0$ raw	0.054	0.99	0.029	0.431	0.183	0.157
vehicle$\times\mu \times v_0$ + across-μ	0.022	0.44	0.014	0.352	0.183	0.077

"vehicle$\times\mu \times v_0$" uses fine Mondrian groups; "+ across-μ" applies conservative smoothing across friction bins. Both reduce run-level availability due to small-support calibration partitions, despite similar FAR_{run} targets.

S6. Physics reference monitor (label-aligned; past-only)

To complement the learned hazard scorer, we instantiate an interpretable physics reference monitor constructed from the same Route-B predicate family used to define t_{ev}. This monitor assumes access to the same vehicle/environment descriptors used by the proxy (vehicle signature parameters and the friction descriptor μ). In this dataset, these quantities are available as run-level metadata; in deployment, they would be provided by configuration or external estimation modules (estimating μ itself is out of scope). Because it is label-aligned, its primary role is as a sanity check and audit artifact (not a fair competing predictor).

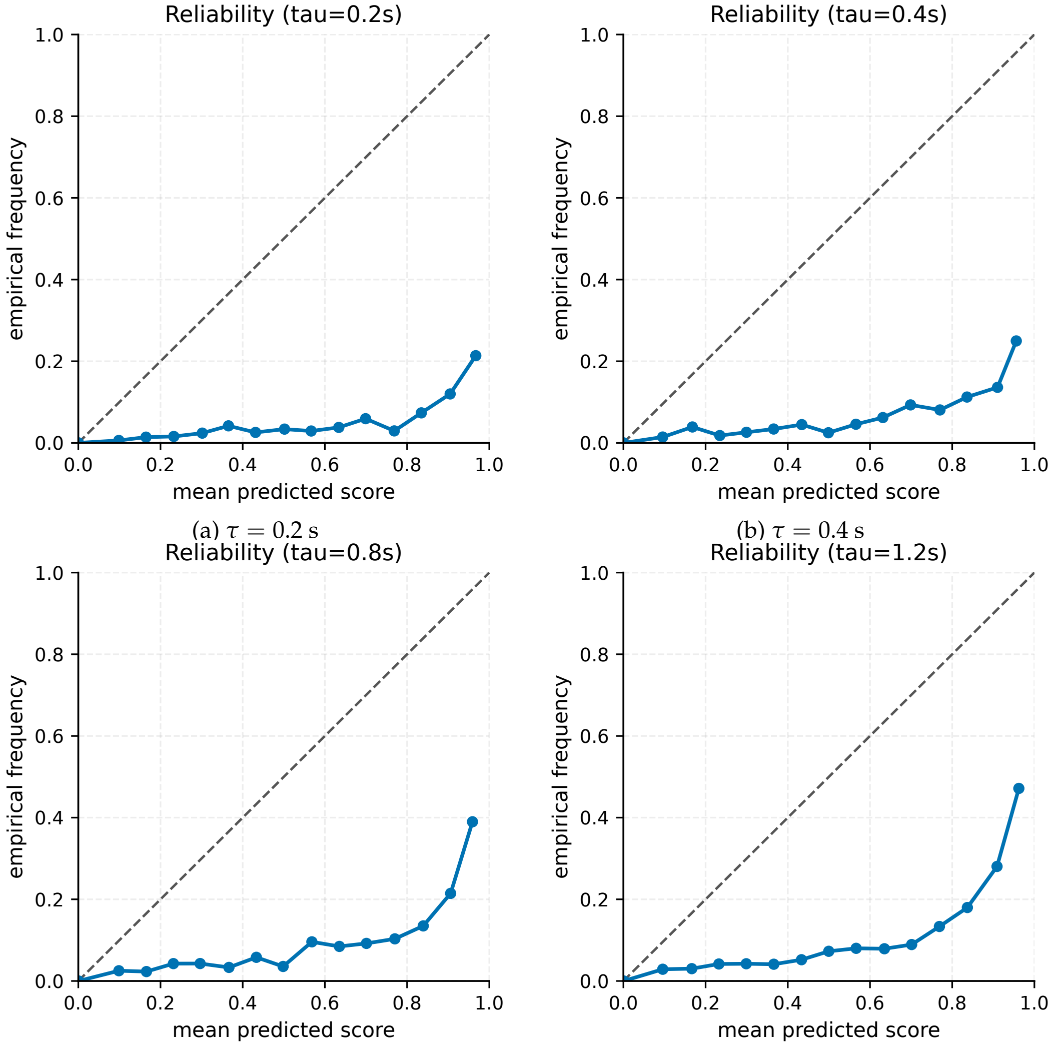

(a) $\tau = 0.2$ s (b) $\tau = 0.4$ s

(c) $\tau = 0.8$ s (d) $\tau = 1.2$ s

Figure S4. Reliability diagrams across horizons (test split).

S7. Path-margin robustness evidence (non-vacuous certificate)

To make the *path-margin* notion defensible, we report a simple robustness evidence package: (i) The *certified core* fraction under a physically motivated feature-noise budget and (ii) Monte Carlo decision flip rates under bounded perturbations. We use feature-wise budgets derived from calibration dispersion, $u_j = \max(\delta_{\min}, \beta\,\sigma_j^{\mathrm{cal}})$ with $\beta = 0.05$ and $\delta_{\min} = 10^{-6}$, and probe normalized radii ϵ in units of these budgets.

S8. Finite-sample distribution-free control (proof sketch)

This section gives a self-contained proof sketch for the one-sided distribution-free false-alarm control used in the paper.

S8.1 Conservative upper quantile

Given a multiset of calibration negative scores $\{s_1, \ldots, s_n\}$ and an index $q \in (0,1)$, define the *upper empirical quantile.*

$$\mathrm{Quantile}_q^{\uparrow}(\{s_i\}) \triangleq \min\Big\{t : \#\{i : s_i \le t\} \ge \lceil qn \rceil\Big\}. \tag{1}$$

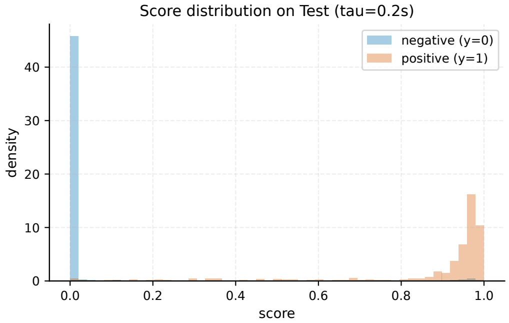

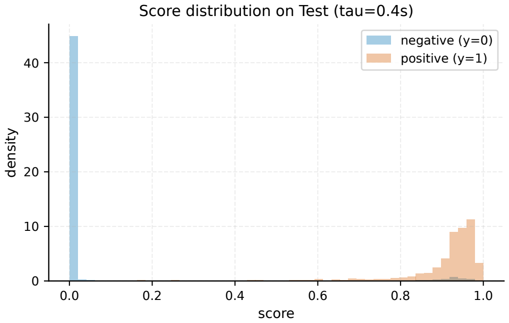

(a) $\tau = 0.2$ s (b) $\tau = 0.4$ s

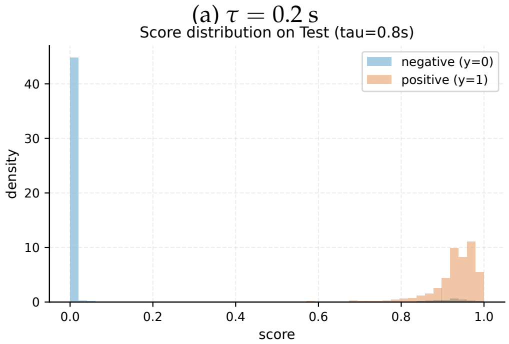

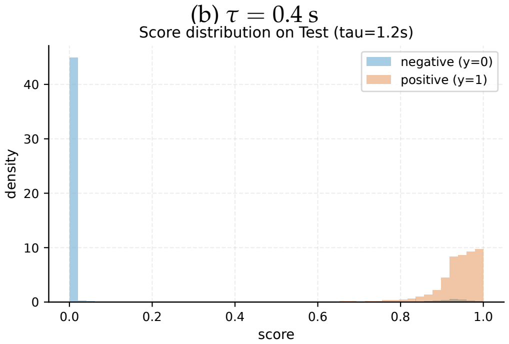

(c) $\tau = 0.8$ s (d) $\tau = 1.2$ s

Figure S5. Score histograms across horizons (Train/Val/Cal/Test).

Table S7. Physics reference monitor (prule) run-level performance at $\tau = 0.2$ s after run-level calibration ($\alpha_{\text{run}} = 5\%$, rolling-mean aggregation $k = 2$).

Monitor	α_{run} (%)	thr	FAR_{run}	FAR_{time} (%)	EWR (all)	Mean lead (s)	EWR (warnable)
prule	5	0.480	0.046	1.01	0.040	0.806	0.217

The prule monitor uses only the conservative physics proxy (Route-B instability predicate) without learned scoring. It attains lower EWR than the learned monitor at similar FAR_{run}, while exhibiting longer mean lead time on the warned subset.

This convention ensures that:

$$\#\{i : s_i > \text{Quantile}_q^{\uparrow}(\{s_i\})\} \le n - \lceil qn \rceil = \lfloor n(1-q) \rfloor.$$

Consequently, the strict alarm rule $s > \text{Quantile}_q^{\uparrow}$ is conservative without any no-ties assumption; if one instead alarms on $s \ge \text{Quantile}_q^{\uparrow}$, then an explicit tie-breaking rule or an additional no-atom assumption is needed.

S8.2 Global split-conformal FPR control

Let $s^\star$ be the score of a *negative* test slice and assume that $(s_1, \ldots, s_n, s^\star)$ are exchangeable. Set the threshold $t_\alpha = \text{Quantile}_{1-\alpha}^{\uparrow}(\{s_i\}_{i=1}^n)$ and alarm only when $s^\star > t_\alpha$. Under exchangeability, after adding infinitesimal i.i.d. tie-breaking jitter (equivalently, any randomized tie rule), the rank of $s^\star$ among the $n+1$ augmented values is uniform on $\{1, \ldots, n+1\}$. Because exact ties at t_α are non-alarms under the strict rule and $t_\alpha = \text{Quantile}_{1-\alpha}^{\uparrow}(\{s_i\}_{i=1}^n)$, the event $\{s^\star > t_\alpha\}$ implies that $s^\star$ ranks among the largest

$$n - \lceil (1-\alpha)n \rceil + 1 = \lfloor n\alpha \rfloor + 1$$

values of the $n+1$ augmented scores; hence:

$$\mathbb{P}(s^\star > t_\alpha) \le \frac{n - \lceil (1-\alpha)n \rceil + 1}{n+1} = \frac{\lfloor n\alpha \rfloor + 1}{n+1} \le \alpha + \frac{1}{n+1}. \tag{2}$$

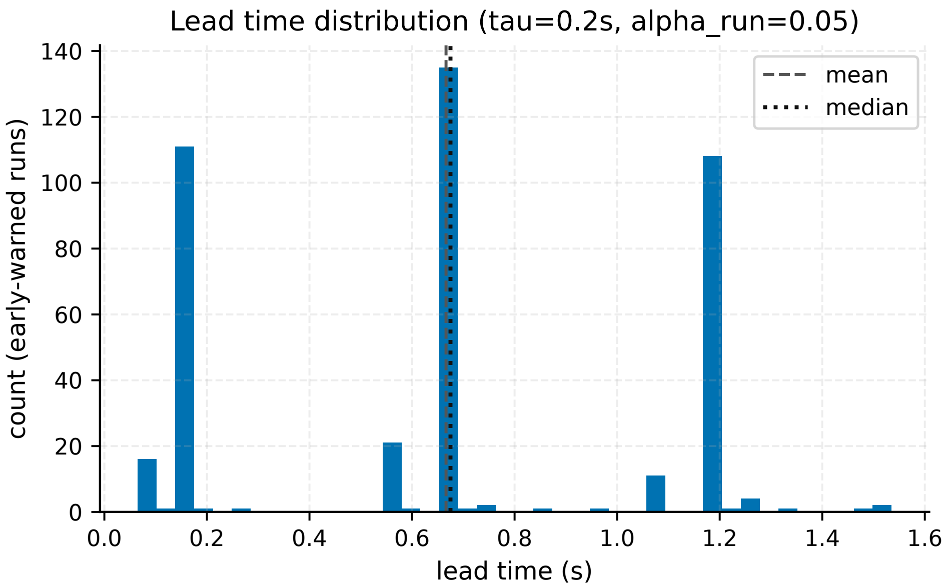

Figure S6. Lead-time distribution for early-warned event runs at $\tau = 0.2$ s under a representative run-level budget $\alpha_{\text{run}} = 5\%$ (rolling-mean aggregation, $k = 2$). Lead time is measured as $t_{\text{ev}} - t_{\text{first alarm}}$.

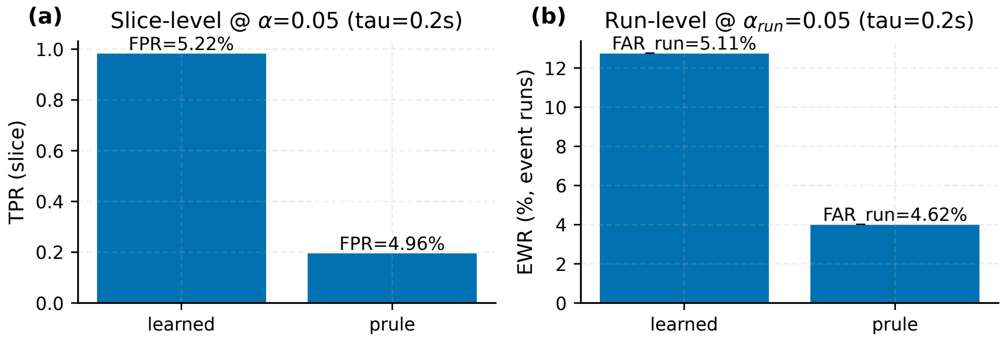

Figure S7. Learned hazard scorer versus physics reference monitor (prule) at $\tau = 0.2$ s and $\alpha = 5\%$ (slice level) / $\alpha_{\text{run}} = 5\%$ (run level). The learned scorer achieves substantially higher slice-level TPR at similar false-alarm budgets, with comparable FAR_{run}.

This yields finite-sample, distribution-free control of the slice-level false positive rate (FPR) on negative slices up to a discretization slack.

S8.3 Mondrian (group-conditional) control

For a group map $g(x)$, apply the same argument within each group g using only the n_g calibration negative scores in that group. If calibration and test negatives are exchangeable *within a group*, then under the same strict-exceedance rule:

$$\mathbb{P}(s^\star > t_\alpha(g) \mid g(x^\star) = g) \le \frac{\lceil (n_g + 1)\alpha \rceil}{n_g + 1}. \tag{3}$$

Hierarchical back-off to a parent threshold (e.g., vehicle → global) preserves the corresponding (coarser) validity level.

S9. Additional robustness and audit experiments

This section provides additional experiments to further interrogate the deployment interface and the assumptions underpinning the distribution-free false-alarm guarantee. Specifically, we report (i) threshold-selection baselines (ROC/Youden and F_1-based) versus the risk-knob interface, (ii) per-vehicle dispersion of realized false-alarm rates under

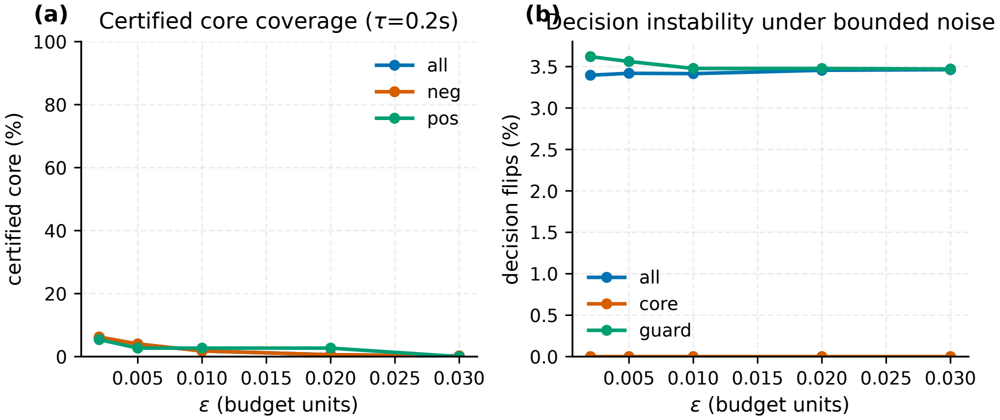

Figure S8. Path-margin robustness at $\tau = 0.2$ s and $\alpha = 5\%$: (a) certified-core coverage versus ϵ and (b) Monte Carlo decision flip rates under bounded perturbations.

Table S8. Path-margin robustness at $\tau = 0.2$ s and $\alpha = 5\%$. We report certified-core coverage $\Pr[\rho(x) > \epsilon]$ and Monte Carlo decision flip rates under bounded perturbations $|\delta x_j| \le \epsilon\, u_j$, where $u_j = \max(\delta_{\min}, \beta\, \sigma_j^{\text{cal}})$ with $\beta = 0.05$ and $\delta_{\min} = 10^{-6}$. We evaluate $n = 5{,}000$ test slices near the calibrated threshold ($|f_\tau(x) - t_\alpha| \le 0.05$) with 30 Monte Carlo draws per slice.

ϵ	Core (all) (%)	Core (neg) (%)	Core (pos) (%)	Flips (all) (%)	Flips (core) (%)	Flips (guard) (%)
0.002	6.20	6.21	5.41	3.40	0.00	3.62
0.005	4.00	4.01	2.70	3.42	0.00	3.56
0.010	1.80	1.79	2.70	3.42	0.00	3.48
0.020	0.60	0.58	2.70	3.46	0.00	3.48
0.030	0.22	0.22	0.00	3.46	0.00	3.47

Across the probed ϵ range, flips are confined to the *guard* set while the certified core exhibits zero observed flips, indicating that the certificate is non-vacuous and that decision instability under bounded noise concentrates near the threshold.

global versus vehicle-conditioned thresholds, (iii) a run-blocked bootstrap calibration that reduces within-run slice dependence in the calibration support, and (iv) a subdomain mismatch stress test illustrating how false-alarm control can drift when exchangeability is intentionally violated.

S9.1 Threshold selection baselines versus the risk knob

A common alternative to risk-budgeted calibration is to select a fixed operating threshold on a development split using ROC heuristics (e.g., the Youden index) or a classification metric (e.g., maximum F_1). Figure S9 and Table S10 compare these choices against split-conformal calibration at $\tau = 0.2$ s. While ROC/F_1 thresholds can yield competitive operating points at a particular mixture, they do not implement an auditable false-alarm budget and can substantially violate a prescribed α (especially at tighter budgets).

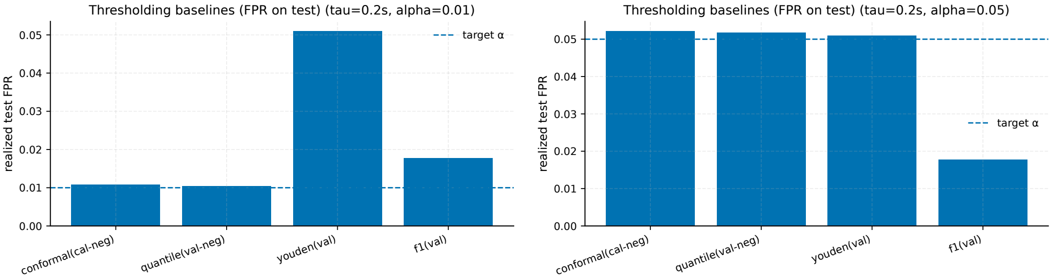

Figure S9. Realized test FPR under several threshold-selection baselines at $\tau = 0.2$ s. Each panel reports the realized FPR on the held-out test negatives when the threshold is chosen by a particular method. Left: target budget $\alpha = 1\%$. Right: $\alpha = 5\%$.

Table S9. Illustrative per-feature uncertainty budgets u_j used for the path-margin robustness analysis at $\tau = 0.2$ s. Budgets are derived from calibration-set dispersion after imputation as $u_j = \max(\delta_{\min}, \beta\,\sigma_j^{\mathrm{cal}})$ with $\beta = 0.05$ and $\delta_{\min} = 10^{-6}$. (A full list for all features is exported by the code as a CSV artifact.)

Feature	σ_j^{cal}	u_j
mu	0.2069	0.01034
v0_kmh	28.09	1.404
vx_mean	7.75	0.3875
ay_mean	3.122	0.1561
r_mean	0.3253	0.01626
beta_mean	0.1616	0.00808
phi_mean	0.02344	0.001172
theta_mean	0.007147	0.0003573

Table S10. Threshold-selection baselines at $\tau = 0.2$ s. We compare split-conformal calibration on calibration negatives (our deployment interface) against common development-set threshold heuristics (negative quantile on Val negatives, Youden index on Val, and maximum-F_1 on Val). Reported FPR/TPR are realized on the held-out test split.

α (%)	Method	Threshold	FPR (test)	TPR (test)
1	conformal(cal-neg)	0.963	0.011	0.511
	quantile(val-neg)	0.964	0.010	0.498
	youden(val)	0.141	0.051	0.981
	f1(val)	0.928	0.018	0.740
5	conformal(cal-neg)	0.124	0.052	0.982
	quantile(val-neg)	0.131	0.052	0.982
	youden(val)	0.141	0.051	0.981
	f1(val)	0.928	0.018	0.740

S9.2 Per-vehicle false-alarm dispersion (global vs. Mondrian)

To make the fleet interface auditable at the platform level, we report a per-vehicle breakdown of realized slice-level rates on the test split. Figure S10 summarizes the distribution of per-vehicle FPR under global versus vehicle-conditioned (Mondrian) thresholds, while Table S11 reports per-vehicle counts and operating points.

S9.3 Run-blocked bootstrap calibration (slice dependence diagnostic)

Split-conformal FPR control is stated under exchangeability of negative slices, while in practice, the calibration set contains multiple highly correlated slices per run. To assess sensitivity to within-run dependence, we perform a run-blocked bootstrap: for each replicate, we construct a calibration negative set by sampling *one* negative slice per calibration run, calibrate the $\alpha = 5\%$ threshold, and evaluate the resulting test operating point. Figure S11 and Table S12 summarize 200 replicates.

S9.4 Subdomain mismatch stress test (intentional non-exchangeability)

Finally, we provide a simple stress test illustrating how false-alarm control can drift when calibration and deployment negatives are not exchangeable. We calibrate thresholds on restricted negative subsets defined by a simple query (low/high friction μ or low/high initial speed v_0) and evaluate on (i) the matched test subset, (ii) the complementary subset, and (iii) all test negatives. Table S13 makes those subset definitions explicit and reports the resulting realized FPRs at $\alpha = 1\%$ and $\alpha = 5\%$ by listing the exact query thresholds, calibration/test support, and descriptor summaries of the matched subsets. Figure S12

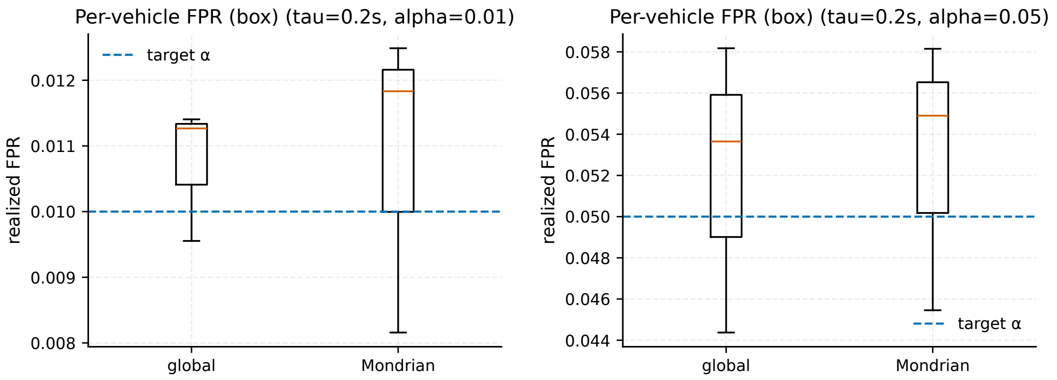

Figure S10. Per-vehicle FPR dispersion at $\tau = 0.2$ s under global versus vehicle-conditioned (Mondrian) calibration. Left: $\alpha = 1\%$. Right: $\alpha = 5\%$.

Table S11. Per-vehicle slice-level operating points at $\tau = 0.2$ s under global versus vehicle-conditioned (Mondrian) calibration, together with the protocol summary for the design ablations. Reported rates are computed on the held-out test split. The back-off fraction is the fraction of test slices for which the vehicle threshold backs off to the global threshold (0 here due to adequate per-vehicle calibration support).

Per-vehicle slice-level operating points at $\tau = 0.2$ s

α (%)	Vehicle	n_0	n_1	$\mathrm{FPR_{glob}}$	$\mathrm{FPR_{veh}}$	$\mathrm{TPR_{glob}}$	$\mathrm{TPR_{veh}}$	back-off
1	`vehicle_1`	60060	388	0.011	0.008	0.487	0.443	0.000
	`vehicle_2`	57336	422	0.011	0.012	0.491	0.536	0.000
	`vehicle_3`	57467	312	0.010	0.012	0.567	0.638	0.000
5	`vehicle_1`	60060	388	0.058	0.045	0.995	0.964	0.000
	`vehicle_2`	57336	422	0.054	0.058	0.976	0.988	0.000
	`vehicle_3`	57467	312	0.044	0.055	0.974	0.990	0.000

Design-ablation protocol summary

Study	Settings	Re-slice	Refit scorer	Recalibrate	Fixed split	Main question
Stride ablation	$d \in \{0.10, 0.05\}$ s with $w = 0.10$ s and $k = 2$	Yes	Yes	Yes	Same run-level split for all settings	How much warning coverage is gained by denser cadence, and at what overlap/workload cost?
Window ablation	$w \in \{0.05, 0.10, 0.20\}$ s with $d = 0.10$ s and $k = 2$	Yes	Yes	Yes	Same run-level split for all settings	How much does shorter/longer past history change fidelity versus warnability?
Aggregation ablation	$k \in \{1, 2, 3\}$ with $w = d = 0.10$ s	No	No	Yes (run-level safe runs only)	Same slice scores and same run-level split	How much chatter suppression is gained from averaging, and how much warnability is lost?

visualizes the same mismatch profiles. These experiments are intentionally mismatched; they do not contradict the conformal guarantee, but illustrate the need for distribution matching and periodic recalibration in deployment.

S10. Design ablations for stride, window length, and run-level aggregation

Table S11 makes the protocol explicit for the design studies in this section. Stride and window ablations rerun the entire slice-level pipeline end-to-end on the same Train/Val/Cal/Test run-level split, whereas the aggregation ablation reuses the same slice scores and changes only the run-level calibration on safe runs.

Stride ablation (0.10 s versus 0.05 s).

We compare the default non-overlapping 0.10 s stride with a 0.05 s overlapping stride while keeping the history window fixed at 0.1 s. Table S14 shows that the denser stride raises $\mathrm{EWR_w}$ substantially, but it also nearly doubles the number of evaluated slices, slightly increases $\mathrm{FAR_{run}}$, and does not improve the mean lead time. We therefore retain the 0.10 s stride as the main deployment configuration and treat 0.05 s as an optional higher-cadence mode.

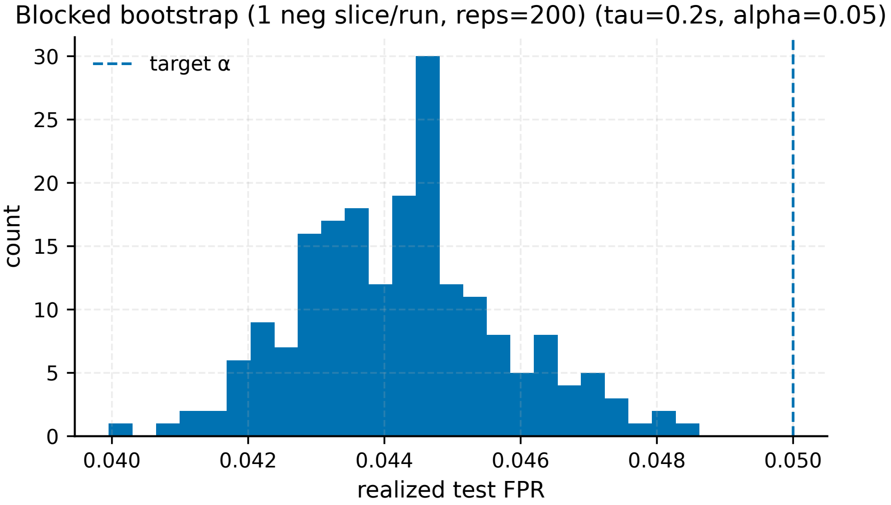

Figure S11. Run-blocked bootstrap calibration at $\tau = 0.2$ s and $\alpha = 5\%$. The histogram shows the distribution of realized test FPR over 200 bootstrap replicates when the calibration set is constructed by sampling one negative slice per calibration run.

Table S12. Run-blocked bootstrap calibration at $\tau = 0.2$ s and $\alpha = 5\%$. Each replicate calibrates the threshold using one negative slice sampled per calibration run (thus reducing within-run slice dependence) and evaluates the resulting operating point on the held-out test split. We report summary statistics over 200 replicates.

Metric	mean	std	min	25%	median	max
t_α	0.250	0.028	0.174	0.234	0.248	0.334
$\mathrm{FPR}_{\mathrm{test}}$	0.044	0.002	0.040	0.043	0.044	0.049
$\mathrm{TPR}_{\mathrm{test}}$	0.965	0.004	0.950	0.963	0.964	0.976

Window-length ablation.

At fixed stride $d = 0.10$ s and aggregation length $k = 2$, Table S14 quantifies how the history window changes the first eligible pre-event aggregated statistic, the warnable rate, and the slice/run-level operating point. A shorter window ($w = 0.05$ s) increases warning feasibility under terminal-phase alignment, but it also slightly worsens slice/run-level false alarms and shortens the mean lead time. A longer window ($w = 0.20$ s) modestly improves slice-level conservatism and mean lead, but it delays eligibility to 0.30 s and therefore loses many warning-feasible runs. The default $w = 0.10$ s is retained as the engineering middle ground.

Run-level aggregation ablation.

Table S14 varies the rolling-mean length at fixed $w = d = 0.10$ s. The $k = 1$ monitor maximizes warnability and overall EWR, but it is also the chattiest configuration, with substantially more repeated alarm onsets on false-alarm safe runs. Increasing to $k = 3$ reduces chatter and $\mathrm{FAR}_{\mathrm{run}}$, but it pushes the first eligible pre-event aggregated statistic to 0.30 s and sharply lowers warnability. The default $k = 2$, therefore, balances run-level smoothness against warning feasibility.

S11. Missing-signal robustness

We stress several representative missing-signal scenarios to assess how the default train-median imputation behaves under deployment-side outages. Table S15 fixes the protocol at the raw 100 Hz channel level and reports the resulting operating points: the "yaw-rate random dropout" row masks 10% of yaw-rate samples independently during

Table S13. Subdomain mismatch stress tests at $\tau = 0.2$ s for $\alpha = 1\%$ and $\alpha = 5\%$, together with the definitions of the restricted negative subsets used for intentional calibration–test mismatch. Thresholds are calibrated on restricted negative subsets and then evaluated on matched versus complementary negative subsets on Test to illustrate potential FPR drift under covariate shift. TPR is undefined (N/A) when a test subset contains no positives.

Stress test results at $\alpha = 1\%$

Cal subset	Test subset	Threshold	n_0^{cal}	n_0^{test}	FPR	TPR	Note
mu_low	match	0.969064	40541	54164	0.011	0.721	
	complement	0.969064	40541	120699	0.007	0.288	
	all	0.969064	40541	174863	0.008	0.430	
mu_high	match	0.961885	47008	63308	0.011	0.343	
	complement	0.961885	47008	111555	0.011	0.624	
	all	0.961885	47008	174863	0.011	0.525	
v0_low	match	0.000038	57780	77220	0.009	N/A	No positives in matched test subset
	complement	0.000038	57780	97643	0.210	1.000	
	all	0.000038	57780	174863	0.121	1.000	
v0_high	match	0.983893	14839	20536	0.014	0.124	
	complement	0.983893	14839	154327	0.000	0.000	
	all	0.983893	14839	174863	0.002	0.121	

Stress test results at $\alpha = 5\%$

Cal subset	Test subset	Threshold	n_0^{cal}	n_0^{test}	FPR	TPR	Note
mu_low	match	0.003560	40541	54164	0.051	1.000	
	complement	0.003560	40541	120699	0.088	0.999	
	all	0.003560	40541	174863	0.077	0.999	
mu_high	match	0.219651	47008	63308	0.052	0.931	
	complement	0.219651	47008	111555	0.042	0.989	
	all	0.219651	47008	174863	0.046	0.969	
v0_low	match	0.000020	57780	77220	0.047	N/A	No positives in matched test subset
	complement	0.000020	57780	97643	0.263	1.000	
	all	0.000020	57780	174863	0.167	1.000	
v0_high	match	0.972812	14839	20536	0.051	0.366	
	complement	0.972812	14839	154327	0.000	0.214	
	all	0.972812	14839	174863	0.006	0.362	

Definitions of restricted negative subsets

Query	Definition	n_0^{cal}	$n_{0,\text{match}}^{\text{test}}$	$n_{0,\text{comp}}^{\text{test}}$	Descriptor summary in matched test subset
`mu_low`	$\mu \leq 0.45$	40541	54164	120699	median $\mu = 0.35$; IQR $[0.30, 0.40]$
`mu_high`	$\mu \geq 0.80$	47008	63308	111555	median $\mu = 0.90$; IQR $[0.85, 0.95]$
`v0_low`	$v_0 \leq 18\,\text{m/s}$	57780	77220	97643	median $v_0 = 14.7\,\text{m/s}$; IQR $[13.2, 16.0]$ m/s
`v0_high`	$v_0 \geq 26\,\text{m/s}$	14839	20536	154327	median $v_0 = 27.9\,\text{m/s}$; IQR $[26.8, 29.7]$ m/s

replay, whereas the "burst" rows correspond to contiguous outages in the named channels rather than isolated missing entries. The "as-is" policy leaves the default train-median imputation and alarm logic unchanged; the "ffill + median" row applies causal forward-fill with train-median fallback when no previous valid value is available; and the "abstain" row suppresses alarm updates whenever the stressed joint channel set is unavailable. Coverage is reported as the fraction of slices for which the monitor remains active under that policy. Isolated 10% random yaw-rate dropout barely changes the interface, but burst dropout on a_y or joint burst dropout on $v_x/a_y/r$ clearly degrades EWR_w. A simple forward-fill policy partially recovers performance relative to leaving the median-imputed baseline unchanged, while an abstaining degraded-mode policy lowers FPR sharply at the cost of coverage and warning effectiveness.

S12. Run-level μ-perturbation sensitivity and no-μ ablation

Because the instability proxy and optional model inputs may depend on the friction descriptor μ, we examine sensitivity to run-level μ perturbation. In the present dataset, μ appears as run-level metadata rather than a within-run dynamic estimate, so the relevant stress tests are bias, noise, coarsening, and omission. Table S16 shows that moderate noise or coarse quantization leaves the operating point close to baseline, while the no-μ feature retrain also remains close to the baseline slice- and run-level operating points. The resulting conclusion is deliberately narrow: these experiments support robustness to moderate run-level μ descriptor perturbation and feature omission, but they do not evaluate a time-varying online friction estimator with within-run lag.

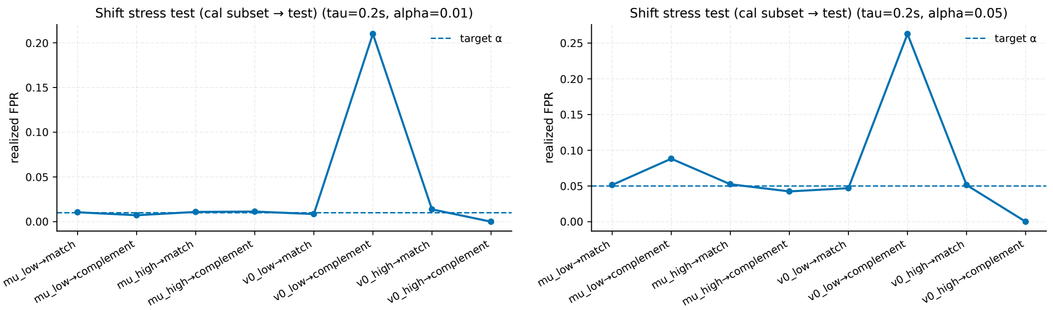

Figure S12. Subdomain mismatch stress test at $\tau = 0.2$ s. Thresholds are calibrated on a restricted negative subset (Cal) and evaluated on matched versus complementary subsets on Test. Left: $\alpha = 1\%$. Right: $\alpha = 5\%$.

Table S14. Design ablations at the primary horizon $\tau = 0.2$ s. The merged table reports the stride, window-length, and run-level aggregation ablations. All settings follow the protocol summarized in Supplementary Table S11.

**Stride ablation (fixed history window $w = 0.10$ s and aggregation length $k = 2$)**									
Stride (s)	Test slices	Slice FPR	Slice TPR	FAR_{run}	Warnable	EWR_w	EWR	Mean lead (s)	Note
0.10	175985	0.052	0.982	0.051	–	0.697	–	0.568	Runtime multiplier = 1.00×
0.05	343448	0.052	0.984	0.053	–	0.806	–	0.534	Runtime multiplier = 1.95×
**Window-length ablation (fixed stride $d = 0.10$ s and aggregation length $k = 2$)**									
Window w (s)	First eligible time (s)	Slice FPR	Slice TPR	FAR_{run}	Warnable	EWR_w	EWR	Mean lead (s)	Note
0.05	0.15	0.055	0.967	0.054	0.259	0.596	0.154	0.503	
0.10	0.20	0.052	0.982	0.051	0.183	0.697	0.127	0.568	
0.20	0.30	0.049	0.975	0.049	0.141	0.709	0.100	0.613	
**Run-level aggregation ablation (fixed $w = d = 0.10$ s)**									
k	First eligible time (s)	Slice FPR	Slice TPR	FAR_{run}	Warnable	EWR_w	EWR	Mean lead (s)	Alarm onsets per false-alarm run
1	0.10	–	–	0.053	0.308	0.561	0.173	0.492	2.34
2	0.20	–	–	0.051	0.183	0.697	0.127	0.568	1.39
3	0.30	–	–	0.048	0.141	0.709	0.100	0.611	1.18

S13. Run-level bootstrap uncertainty

Table S17 reports bootstrap confidence intervals using runs as the resampling unit and warned event runs as the unit for lead-time uncertainty. At $\alpha_{run} = 5\%$, the intervals confirm that FAR_{run} remains close to the target budget while EWR_w and mean lead time remain comparatively stable. As in the main text, lead time is measured as $t_{ev} - t_{first\,alarm}$.

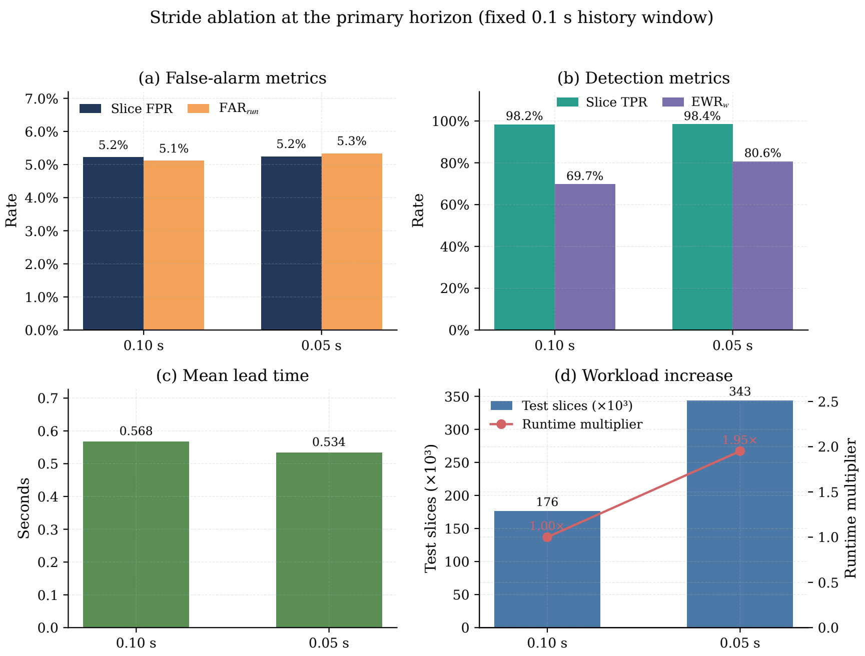

Figure S13. Stride ablation at the primary horizon. Panels summarize false-alarm metrics, detection metrics, mean lead time, and workload increase when the stride is reduced from 0.10 s to 0.05 s at a fixed 0.1 s history window.

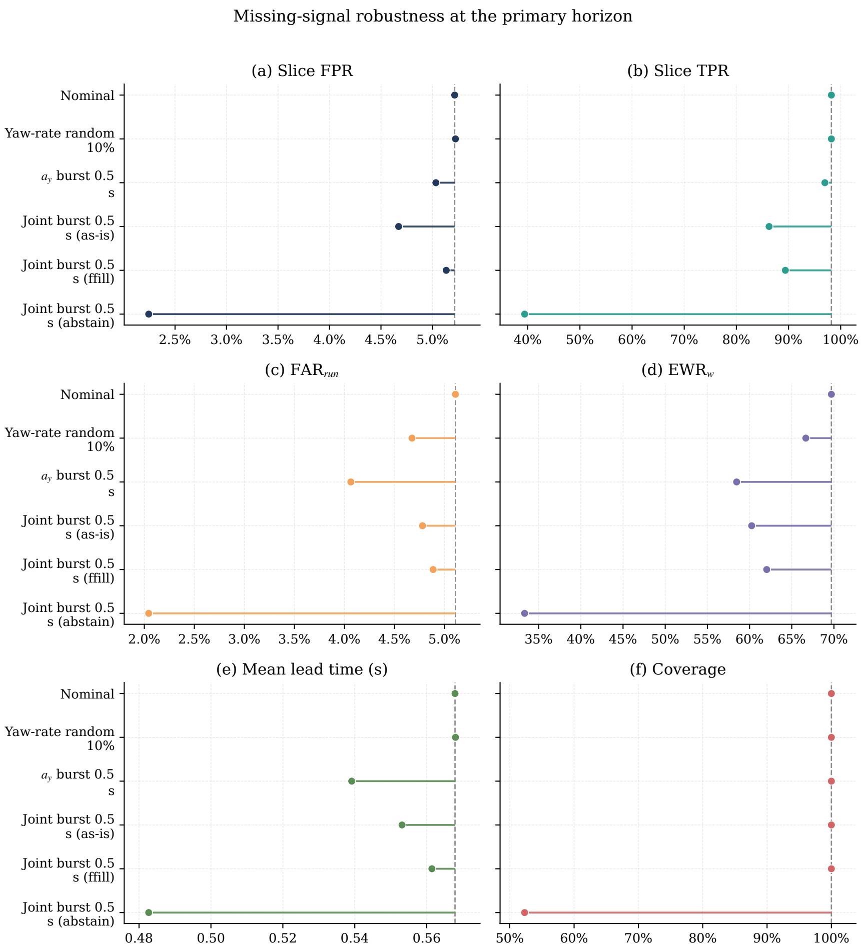

Figure S14. Missing-signal robustness at the primary horizon. Each panel reports one deployment metric relative to the nominal pipeline under random dropout, burst dropout, forward-fill recovery, and abstaining degraded mode.

Table S15. Missing-signal robustness at $\tau = 0.2$ s and $\alpha = 5\%$ / $\alpha_{\text{run}} = 5\%$, together with the protocol definitions for the corresponding stress tests. Corruption is injected at the raw 100 Hz channel level before slice feature extraction. "Yaw-rate random dropout" masks 10% of yaw-rate samples independently; "burst" denotes contiguous outages in the named channels; "as-is" keeps the default train-median imputation and alarm logic unchanged; "ffill + median" applies causal forward-fill with train-median fallback; and "abstain" suppresses alarm updates when the stressed joint channel set is unavailable. Coverage is the fraction of slices for which the monitor remains active under the given policy.

Missing-signal robustness results

Scenario	Policy	Slice FPR	Slice TPR	FAR_{run}	EWR_{w}	Lead (s)	Coverage	Note
Nominal pipeline	baseline	0.052	0.982	0.051	0.697	0.568	1.000	
Yaw-rate random dropout	as-is	0.052	0.982	0.047	0.667	0.568	1.000	
a_y burst dropout	as-is	0.050	0.970	0.041	0.585	0.539	1.000	
Joint $v_x/a_y/r$ burst	as-is	0.047	0.863	0.048	0.602	0.553	1.000	
Joint $v_x/a_y/r$ burst	ffill + median	0.051	0.894	0.049	0.620	0.561	1.000	
Joint burst + abstain	abstain	0.022	0.394	0.020	0.333	0.483	0.523	Alarm updates suppressed during unobservable periods

Protocol definitions for missing-signal stress tests

Scenario label	Channels	Corruption model	Parameters	Placement / evaluation rule
Yaw-rate random dropout	r	Independent Bernoulli masking	$p = 0.10$ per raw sample	Applied over the full 100 Hz replay; monitor policy = `as-is` unless noted otherwise.
a_y burst dropout	a_y	One contiguous outage	$B = 20$ frames $= 0.20$ s; one burst per stressed run	Burst start drawn uniformly from eligible indices that keep the full burst inside the recorded pre-event segment (or inside the safe run).
Joint $v_x/a_y/r$ burst	v_x, a_y, r	One synchronous contiguous outage across all three channels	$B = 30$ frames $= 0.30$ s; one burst per stressed run	Same placement rule as above; evaluated under `as-is`, `ffill + median`, and `abstain` policies.
Forward-fill recovery	stressed channels only	Causal carry-forward with train-median fallback	Previous valid raw sample carried within the burst; median used only if no prior valid sample exists	Applied only after corruption injection; no backward fill is allowed.
Abstaining degraded mode	stressed joint channel set	Alarm suppression during unobservable periods	Alarm updates withheld whenever any of $\{v_x, a_y, r\}$ required by the stressed policy is unavailable in the current update buffer	Coverage is reported as the fraction of slices for which the monitor remains active.

Table S16. Sensitivity to run-level μ perturbation at the primary horizon $\tau = 0.2$ s. Because μ is stored as run-level metadata in the present dataset, we evaluate bias, noise, coarsening, and a no-μ feature ablation rather than a time-varying online estimator with within-run lag.

Scenario	Perturbation	Slice FPR	Slice TPR	FAR_{run}	EWR_{w}	Note
Nominal μ input	0	0.052	0.982	0.051	0.697	baseline
Biased μ estimate	$+0.05$	0.052	0.982	0.051	0.697	constant offset
Biased μ estimate	-0.05	0.056	0.984	0.058	0.763	constant offset
Noisy μ estimate	$\sigma = 0.05$	0.054	0.983	0.055	0.734	Gaussian noise
Noisy μ estimate	$\sigma = 0.10$	0.054	0.984	0.054	0.725	Gaussian noise
Coarsened μ estimate	$q = 0.10$	0.052	0.982	0.051	0.697	quantized input
Coarsened μ estimate	$q = 0.20$	0.054	0.982	0.056	0.743	quantized input
Without μ feature	drop μ	0.052	0.982	0.051	0.684	global retrain without μ

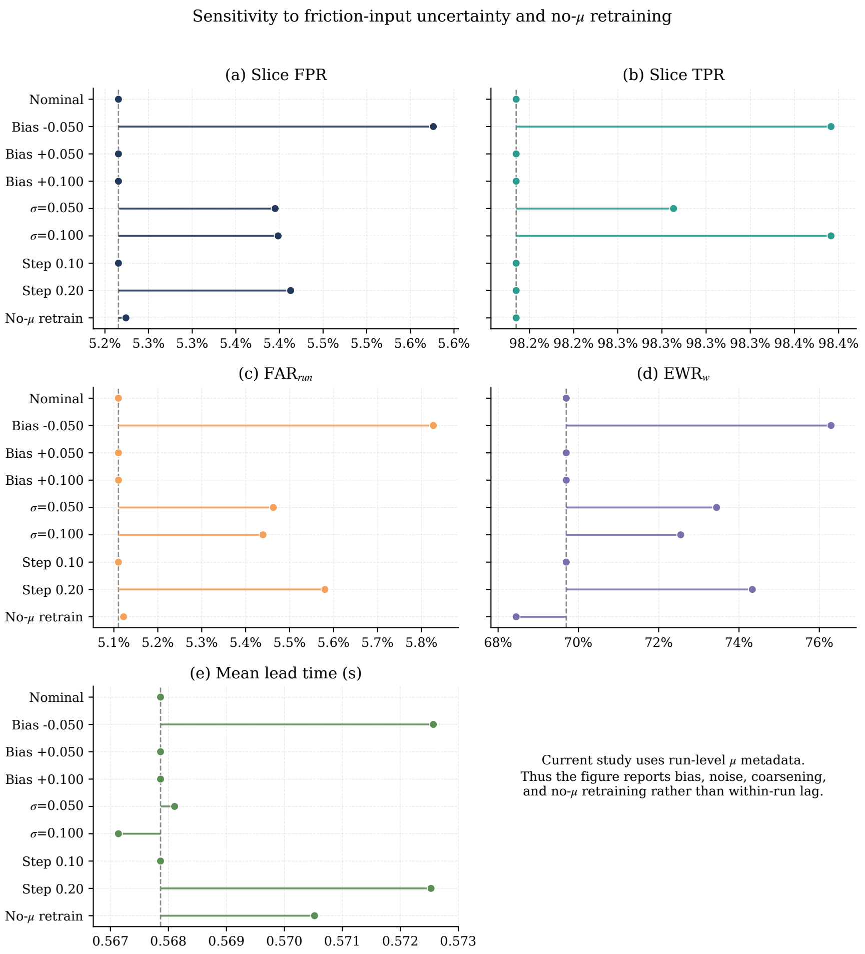

Figure S15. Sensitivity to run-level μ perturbation and no-μ retraining at the primary horizon. Panels summarize slice- and run-level metrics under bias, noise, coarsening, and feature omission; the study does not represent a time-varying within-run estimator.

Table S17. Run-level bootstrap uncertainty bands (1000 replicates). Lead time is measured as $t_{\text{ev}} - t_{\text{first alarm}}$ on warned event runs.

Metric	Point estimate	95% CI	Notes
FAR_{run} @ $\alpha_{\text{run}} = 1\%$	0.012	[0.010, 0.015]	safe run
FAR_{run} @ $\alpha_{\text{run}} = 5\%$	0.051	[0.047, 0.056]	safe run
EWR_{w} @ $\alpha_{\text{run}} = 5\%$	0.697	[0.658, 0.737]	event run
Overall EWR @ $\alpha_{\text{run}} = 5\%$	0.127	[0.116, 0.140]	event run
Lead time (s) @ $\alpha_{\text{run}} = 5\%$	0.568	[0.528, 0.607]	warned event run

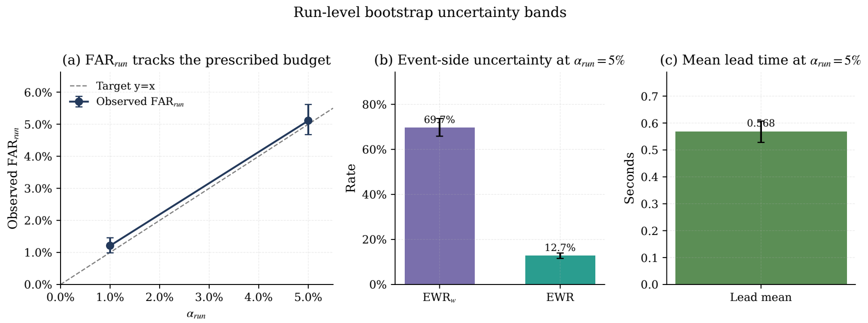

Figure S16. Run-level bootstrap uncertainty bands. Panel (a) compares observed FAR_{run} with the prescribed budget, while panels (b) and (c) summarize event-side uncertainty at $\alpha_{\text{run}} = 5\%$ for EWR_{w}, overall EWR, and mean lead time.
